# A high-contiguity *Brassica nigra* genome localizes active centromeres and defines the ancestral *Brassica* genome

**DOI:** 10.1038/s41477-020-0735-y

**Published:** 2020-08-10

**Authors:** Sampath Perumal, Chu Shin Koh, Lingling Jin, Miles Buchwaldt, Erin E. Higgins, Chunfang Zheng, David Sankoff, Stephen J. Robinson, Sateesh Kagale, Zahra-Katy Navabi, Lily Tang, Kyla N. Horner, Zhesi He, Ian Bancroft, Boulos Chalhoub, Andrew G. Sharpe, Isobel A. P. Parkin

**Affiliations:** 1 Agriculture and Agri-Food Canada, Saskatoon, Saskatchewan Canada; 2 Global Institute for Food Security, University of Saskatchewan, Saskatoon, Saskatchewan Canada; 3 Department of Computing Science, Thompson Rivers University, Kamloops, British Columbia Canada; 4 Department of Mathematics and Statistics, University of Ottawa, Ottawa, Ontario Canada; 5 National Research Council Canada, Saskatoon, Saskatchewan Canada; 6 Department of Biology, University of York, York, UK; 7 Institute of Crop Science, Zhejiang University, Hangzhou, China

**Keywords:** Plant genetics, Next-generation sequencing

## Abstract

It is only recently, with the advent of long-read sequencing technologies, that we are beginning to uncover previously uncharted regions of complex and inherently recursive plant genomes. To comprehensively study and exploit the genome of the neglected oilseed *Brassica nigra*, we generated two high-quality nanopore de novo genome assemblies. The N50 contig lengths for the two assemblies were 17.1 Mb (12 contigs), one of the best among 324 sequenced plant genomes, and 0.29 Mb (424 contigs), respectively, reflecting recent improvements in the technology. Comparison with a de novo short-read assembly corroborated genome integrity and quantified sequence-related error rates (0.2%). The contiguity and coverage allowed unprecedented access to low-complexity regions of the genome. Pericentromeric regions and coincidence of hypomethylation enabled localization of active centromeres and identified centromere-associated ALE family retro-elements that appear to have proliferated through relatively recent nested transposition events (<1 Ma). Genomic distances calculated based on synteny relationships were used to define a post-triplication *Brassica-*specific ancestral genome, and to calculate the extensive rearrangements that define the evolutionary distance separating *B. nigra* from its diploid relatives.

## Abstract (web summary)

Two high-quality nanopore genome assemblies of *Brassica nigra* are reported, one of which has particularly high contiguity with a contig N50 of 17.1 Mb, allowing localization of active centromeres and reconstruction of the ancestral *Brassica* genome.

## Main

Decoding complete genome information is vital for understanding genome structure, providing a full complement of both the genic and repeat repertoire and uncovering structural variation. Such information also provides a foundational tool for crop improvement to facilitate the rapid selection of agronomically important traits and to exploit modern breeding tools such as genome editing^1–3^. Whole-genome duplication and abundant repeat expansion has led to an approximate 660-fold variation in genome size among angiosperms^4^ and, in particular, the low complexity of repetitive regions, including centromeric, pericentromeric and telomeric regions, creates challenges for complete genome assembly using short-read (SR) sequence data^5^. Centromeres are of particular interest due to their biological importance, yet resolving their structure has been frustrated by the prevalence of repetitive elements; commonly these are marked by the presence of short, tandemly repeated sequences and, although similar to one other very small plant genome^6^, no such sequence has been identified for *Brassica nigra*^7,8^.

Recent advances in long-read (LR) sequencing technologies, such as Pacific Biosciences (PacBio) and Oxford Nanopore Technology (ONT)^9^, combined with genome scaffolding methods such as optical mapping and chromosome conformation capture (Hi-C), have led to a paradigm shift in our ability to obtain complete and contiguous genome assemblies^9–11^. Both approaches can produce remarkably long reads, although the error rate is markedly higher than more accurate Illumina short reads, which until recently limited their use to scaffolding in improving assembly contiguity^12^. However, correction algorithms to reduce error rates and recent technological improvements have increased the output and quality of LR sequence data, making possible the routine and cost-effective assembly of large eukaryotic genomes^13^.

The Brassicaceae is an important plant family with approximately 3,800 species including commercially important vegetable, fodder, oilseed and ornamental crops. The Brassiceae tribe has a history of extensive whole-genome duplication events, including the *Brassica* genus-specific whole-genome triplication (WGT), which occurred approximately 22.5 million years ago (Ma) (ref. ^14,15^) and is assumed to be shared by the three important diploids (*Brassica rapa*, AA, 2*n* = 2*x* =20; *B. nigra*, BB, 2*n* = 2*x* = 16; and *Brassica oleracea*, CC, 2*n* = 2*x* = 18) that form the vertices of U’s triangle^16^. Among these three, *B. nigra* (B genome) has been neglected with regard to both genetic analyses and selection through breeding. Due to its limited domestication and its production as out-crossing populations, it has retained valuable allelic diversity compared to its relatives, making it an untapped repository for *Brassica* breeding^17^. Among the six species of U’s triangle, five have been sequenced including, most recently, *B. nigra*, but the assemblies cover at most 80% of the estimated genome size and almost all were very highly fragmented due to the sole use of SR^18–22^. Recently the *B. rapa* reference genome was improved using PacBio sequencing^21^, and one genotype each of *B. rapa* and *B. oleracea* was sequenced using a combination of ONT and optical maps, demonstrating the use of these technologies for complex duplicated genomes^23^.

The work described represents the near-complete assembly of two *B. nigra* genomes (Ni100 and CN115125) using a combination of ONT sequencing, Hi-C and genetic map-based scaffolding. A SR assembly of Ni100 allowed comprehensive benchmarking of the LR assemblies. Remarkably, direct methylome profiling using the ONT data allowed the resolution of candidate active centromeres of the chromosomes, a feature previously unannotated in SR assemblies. In addition, computationally defined genomic distances between the three *Brassica* diploid genomes allowed the construction of an ancestral *Brassica-*specific genome.

## Results

A combination of nanopore sequencing, Illumina error correction, Hi-C sequencing and genetic mapping was used to generate two de novo assemblies for the diploid *Brassica* species, *B. nigra* (genotypes Ni100 and CN115125). Identical sequential steps were followed to assemble the contigs for each genome, including the development of high-quality sequencing datasets, genome assembly and polishing with SR (Supplementary Fig. 1). After testing a number of published assembly software pipelines (Supplementary Table 1), the final contigs were derived from SMARTdenovo using 30–64× coverage of CANU^24^-corrected reads.

**Fig. 1:**
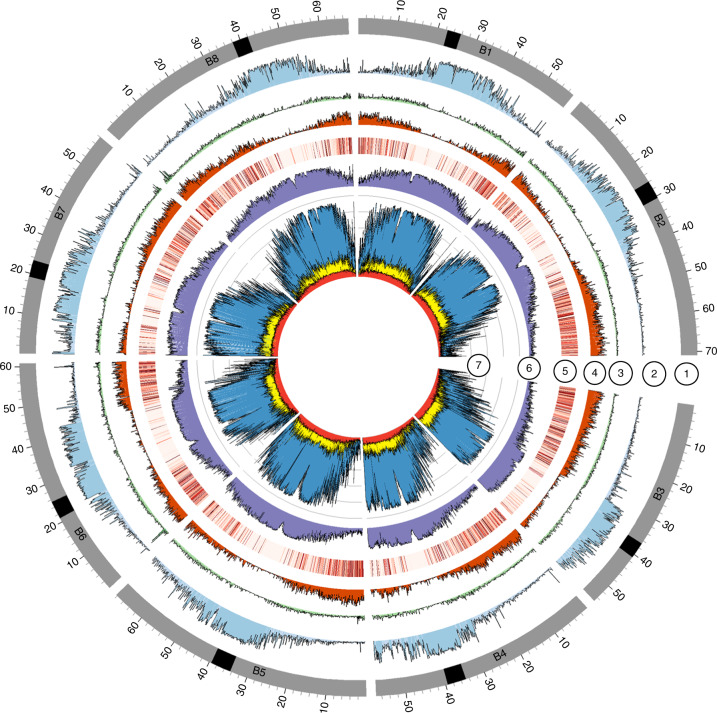
Genomic features of the *B. nigra* Ni100-LR assembly. Bands: (1) chromosomes with centromere positions (black band); (2) class I retrotransposons (nucleotides per 100-kb bins); (3) class II DNA repeats (nucleotides per 100-kb bins); (4) gene density (genes per 100-kb bins); (5) gene expression in leaf tissue (log_10_[average TPM] in 100-kb bins); (6) ONT CG methylation profile (ratio per 100 kb); (7) whole-genome bisulfite methylation profile (nucleotides per 100-kb bins). CG, blue; CHG, yellow; CHH, red.

Although largely context dependent, nanopore sequence data can show error rates up to 15%. Thus, sequence correction was completed using eight rounds of Pilon^25^ with approximately 100× coverage of Illumina data, and quality was assessed at each round through benchmarking universal single-copy orthologue (BUSCO)^26^ scores and qualimap^27^ (Supplementary Fig. 2 and Supplementary Table 2). For both genotypes the read alignment rate was high (>98%) and both tools indicated a significant improvement after correction, suggesting final error rates of between 0.8% (CN115125) and 0.2% (Ni100) at the base pair (bp) level. The two LR assemblies were generated over a period of approximately 12 months, during which time ONT upgraded their library construction kits, pore chemistry and base-calling software. The combined impact of this was noted in an overall improvement in quality, average read length and useable data output for Ni100 and in final assembly contiguity (Supplementary Tables 3–5). Because the CN115125 assembly was more fragmented (compare a contig N50 length of 0.288 Mb with 17.1 Mb), scaffolding using proximity ligation, a combination of Chicago and Hi-C, was used to improve contiguity by up to 193-fold, with a final N50 length of 55.7 Mb (Supplementary Fig. 3). In both instances genetic anchoring was used to generate the final chromosome-scale assemblies of the two *B. nigra* genotypes, CN115125 (C2-LR) and Ni100 (Ni100-LR) (Table 1, Fig. 1, Supplementary Fig. 4 and Supplementary Table 6).

**Table 1:** Statistics of the *B. nigra* genome SR and LR assemblies

Assembly	YZ12151-SR^a^	Ni100-SR	Ni100-LR	C2-LR
Estimated genome size (*k* = 17) (Mb)	591	570	570	608
Assembly size (Mb)	397	447	506	537
No. of chromosomes	8	8	8	8
Genome coverage	0.68	0.78	0.89	0.88
No. of sequences	2,546	19,203	58	963
Longest scaffold (Mb)	45	53	50	71
Scaffold N50 (Mb (no.))	39 (5)	43.9 (5)	60.8 (4)	55.7 (5)
Contig N50 (kb (no.))	38	48 (2,256)	17,127 (12)	288 (424)
Ambiguous bases ‘N’ (kb)	47,528	33,737	13	390
BUSCO (percentage complete)	NA	97	97	94.4
Genomic copy content (%)	38	38	38	38
No. of genes	47,953	56,331	59,877	67,030
High-confidence genes (no. (%))^b^		55,141 (98)	57,798 (97)	64,071 (96)
Low-confidence genes (no. (%))^b^		1,190 (2)	2,079 (3)	2,959 (4)
Repeats and TE space (Mb (%))	134 (33)	183 (41)	273 (54)	263 (49)
Uncharacterized genome (Mb (%))	194 (33)	123 (22)	64 (11)	71 (12)

^a^Information obtained from ref. ^22^; however, the repeat composition was based on the presented analyses being comparable across genomes. NA, not applicable due to not being provided in the reference.

^b^Described in Methods.

**Fig. 2:**
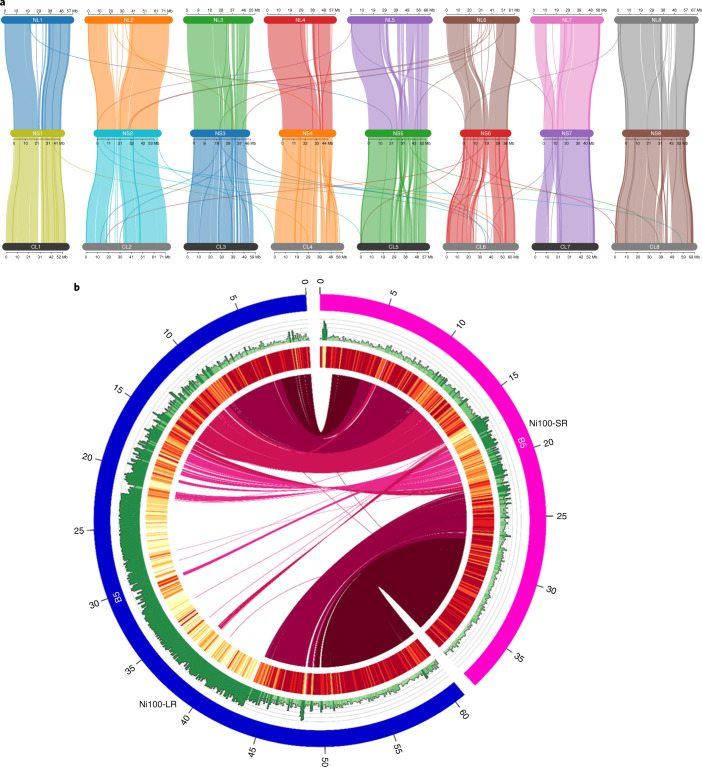
Comparison of *B. nigra* assemblies. **a**, Chromosome-level genome alignment of the Ni100-SR (NS) assembly (centre) against the LR assemblies, C2-LR (bottom) and Ni100-LR (top). The plot was created using Synvisio (https://github.com/kiranbandi/synvisio). **b**, Circular map generated using Circos^89^ showing the alignment of the SR and LR assemblies for chromosome B5 of Ni100.

A SR Illumina de novo assembly for *B. nigra* genotype Ni100 (Ni100-SR) was used for further validation of the nanopore assemblies. The Ni100-SR assembly has a total length of 446.5 Mb from 19,203 scaffolds, of which 367.2 Mb was anchored to eight pseudo-chromosomes (Table 1 and Supplementary Table 6). Alignment and visualization of corresponding pseudo-chromosome sequences from the three *B. nigra* assemblies revealed high levels of collinearity (Fig. 2a). Such high-level comparisons can elucidate large-scale chromosome rearrangements and a number of translocations and inversions were noted—in particular, a large inversion at the bottom of B4 distinguished the SR assembly (Supplementary Fig. 5). This region on B4 was difficult to scaffold in the SR assembly due to limited recombination and shorter scaffold lengths; for such regions in the SR assembly, the order was largely inferred based on synteny data from *Arabidopsis thaliana*. It was apparent that there was expansion of the ONT assemblies in regions presumed to be pericentromeric, as shown in Fig. 2. The level of coverage of these regions also varied between the LR assemblies, with Ni100-LR having the highest.

Gene annotation from the two LR and two SR assemblies (Ni100-SR and the previously published YZ12151 (ref. ^22^)) were rationalized to generate a final *B. nigra* gene complement of 67,030 and 59,877 gene models in the two genotypes, CN115125 and Ni100, respectively. These numbers are in line with the predicted pan-gene content of the diploid *B. oleracea*, with 63,865 ± 31 genes^28^. An additional 3,546 genes were annotated in Ni100-LR compared to the Ni100-SR assembly. A homology search performed using GMAP^29^ (minimum identity and coverage of 95%) indicated that only 914 of the additional genes were unique to the Ni100-LR assembly (Supplementary Fig. 6a). This discrepancy was due both to co-assembly of highly similar genes in the SR data and assembly errors that precluded accurate gene annotation. Read mapping of Illumina data back to the SR and LR assemblies showed a marked increase of 9% multi-mapping reads in the latter with a concomitant reduction in non-concordant matches, suggesting the resolution of duplicated or highly homologous sequences in the LR assembly (Supplementary Table 7). The recent ONT assemblies of *B. rapa* and *B. oleracea* studied the self-incompatibility locus or the S locus region which, due to its repetitive structure, has been notoriously difficult to assemble, to infer the enhanced contiguity of the LR-derived genome sequences^30^. The S locus region was identified and compared in the two *B. nigra* LR assemblies, showing complete assembly of two differing S locus haplotypes (Supplementary Fig. 7). A comparison between the two ONT assemblies would have been expected to identify such genotype differences. Along with approximately 10% of the annotated genes being specific to either assembly, the CN115125 genotype showed a higher prevalence of tandemly and proximally duplicated genes (Supplementary Fig. 6c and Supplementary Tables 8, 9 and 10).

To investigate global gene content across the genomes, annotated genes from the three representations of *B. nigra* were clustered with genes from *B. rapa*, *B. oleracea* and *A. thaliana* using Orthofinder^31^ (Supplementary Figs. 6d and 8a and Supplementary Table 11). The diploid *Brassica* species ranged in number of species-specific genes, with *B. rapa* containing the least while *B. nigra* contained the most (Supplementary Fig. 8b,c and Supplementary Table 11). Sixty-nine of *B. nigra-*specific gene families were deemed to be rapidly evolving by CAFE^32^, and functional analyses demonstrated that the genes were enriched in response to abiotic or biotic stresses, structural molecule activity and unknown molecular functions (Supplementary Fig. 9). Since it is often noted that families related to stress are more prone to differential copy number variation, differences in R genes, transcription factors (TFs) and protein-kinase families were assessed in each of the genomes. The distribution of R gene families across the species appeared to be directly related to genome size and/or expansion of the transposable element complement, with the larger genomes of *B. oleracea* and *B. nigra* showing the greatest expansion of R genes irrespective of genotype (Supplementary Table 12). CN115125 in particular showed increased membership of TF families, with both *B. nigra* genotypes showing a higher prevalence of B3, C2H2 and NAC domain TFs compared to their diploid relatives (Supplementary Tables 13 and 14).

Beyond the large chromosomal rearrangements, structural variations (SVs) in the range of 100 bp to a few Mb, including deletions, insertions, duplications, inversions and translocations that differentiate genotypes, were catalogued between both genomes using ONT reads. The raw ONT reads from Ni100 and CN115125 were aligned to both LR assemblies, and SVs were quantified using two different SV callers (Sniffles^33^ and Picky^34^). Self-alignment was used to estimate a false-positive rate for each genome, which was higher for the CN115125 assembly (6,307 versus 2,230 events) (Supplementary Table 15 and Supplementary Fig. 10). High-quality SVs were considered to be those identified with both software packages (Supplementary Table 15). Dependent on direction of comparison, approximately 6,000–7,000 SVs differentiated the two genotypes, with deletions being the most prevalent (between 63.4 and 70% of events). A small number of deletions were coincident with gene annotations, affecting between 865 and 638 genes (Supplementary Table 16), while a notable proportion was found proximal to genes and thus might have been anticipated to impact expression and/or gene copy number (Supplementary Fig. 10b,c). A set of 161 and 136 SVs in the two genomes were found to overlap completely with annotated full-length, functional, transposable elements, suggesting a mechanism for their formation (Supplementary Table 17).

A *Brassica* B genome-specific repeat library with 1,324 families was developed using multiple annotation tools and was used to survey the repetitive genome fraction of the LR (Ni100-LR, C2-LR) and SR *B. nigra* assemblies (Ni100-SR and YZ12151-SR) (Supplementary Table 18). Repeats spanned 49 and 54% of the CN115125 and Ni100-LR genome assemblies, respectively, compared to 33% (YZ12151) and 41% (Ni100) in the two SR assemblies. The increase in repeat content of the LR assemblies, which closely mirrors the increase in genome captured, predominantly resulted from a rise in annotated class I transposons, in particular Gypsy and Copia elements, which increased by 8% (79 versus 130 Mb) and 4.1% (26 versus 51 Mb), respectively, in the Ni100-LR assembly compared to the Ni100-SR assembly (Supplementary Table 18). The distribution of repeats revealed that class I retrotransposons were more common in traditionally heterochromatic regions such as centromeric, pericentromeric and subtelomeric regions, while class II DNA transposons were more evenly distributed across the genome (Fig. 1 and Supplementary Fig. 4). The identification of centromere- and telomere-specific repeats suggested that ONT assemblies provide more complete access to the chromosome structure (Supplementary Fig. 11). The repeat fraction appears to reflect the estimated genome size of the diploid *Brassica*s, with *B. nigra* lying between *B. oleracea* (~60%)^23^ and *B. rapa* (~38%)^21,23^.

**Fig. 3:**
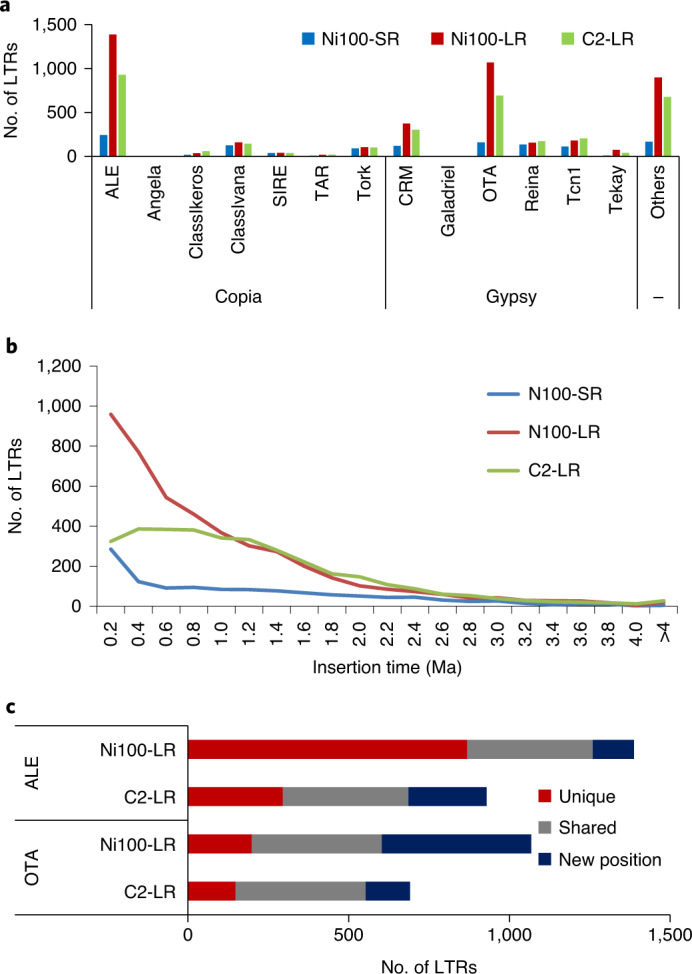
Annotation of FL-LTR-RTs in *B. nigra* genomes. **a**, Copy number of FL-LTR-RTs from 14 different families. **b**, Age distribution of FL-LTR-RTs in three *B. nigra* assemblies. **c**, Comparison of insertion sites of two FL-LTR-RTs (ALE and OTA) in the ONT assemblies. Source data

Almost all families were similarly distributed in the two LR assemblies apart from LTR-Gypsy elements, which were ~5% higher in Ni100, suggesting either Ni100-specific amplification or better assembly of these elements (Supplementary Table 18). Full-length long terminal repeat retrotransposons (FL-LTR-RTs) were annotated and compared in Ni100-SR and the two LR assemblies. A total of 1,220, 4,491 and 3,381 FL-LTR-RTs were identified in Ni100-SR, Ni100-LR and C2-LR assemblies, respectively, with an average length of ~6 kb (Supplementary Table 19 and Supplementary Fig. 12a). The increased annotation of such elements in the LR assemblies indicates the benefits of the technology in regard to assemblage of low-complexity, redundant sequences. Based on repeat domain protein homology, the FL-LTR-RTs were grouped into 14 different families where 41–44% had homology with known Gypsy families, 38–42% with Copia families and 13–20% were unknown FL-LTR-RTs (Fig. 3a). Notably, among the 14 FL-LTR-RT families, members of the ALE (Copia) and OTA (Gypsy) families were specifically increased in copy number in the LR assemblies and more so in the Ni100-LR assembly (Fig. 3a, Supplementary Table 19 and Supplementary Fig. 12a).

The age distribution analysis of FL-LTR-RT elements, based on divergence of the LTR region, showed recent amplification of LTRs in both genomes. About 91% (4,068) and 86% (2,912) of FL-LTR-RTs in Ni100-LR and C2-LR assemblies, respectively, were amplified <2 Ma (Fig. 3b, Supplementary Figs 12b and 13 and Supplementary Table 19), with more recent and continuous proliferation of LTRs (3,056, 68%) aged <1 Ma in Ni100-LR compared to C2-LR. ALE family elements showed more recent amplification (<0.2 Ma) in Ni100-LR compared to C2-LR, while OTA LTRs showed a more consistent pattern between the two genomes. Analysis of the insertion sites revealed that 405 OTA (59%) and 391 ALE (42%) were conserved in both genomes and, in line with the increase in density, there was a higher percentage of uniquely inserted ALE elements in the Ni100-LR genome compared to the C2-LR genome (Fig. 3c). A phylogenetic analysis of ALE and OTA FL-LTRs suggested Ni100 genotype-specific amplification of particular members of each family (Supplementary Fig. 14).

**Fig. 4:**
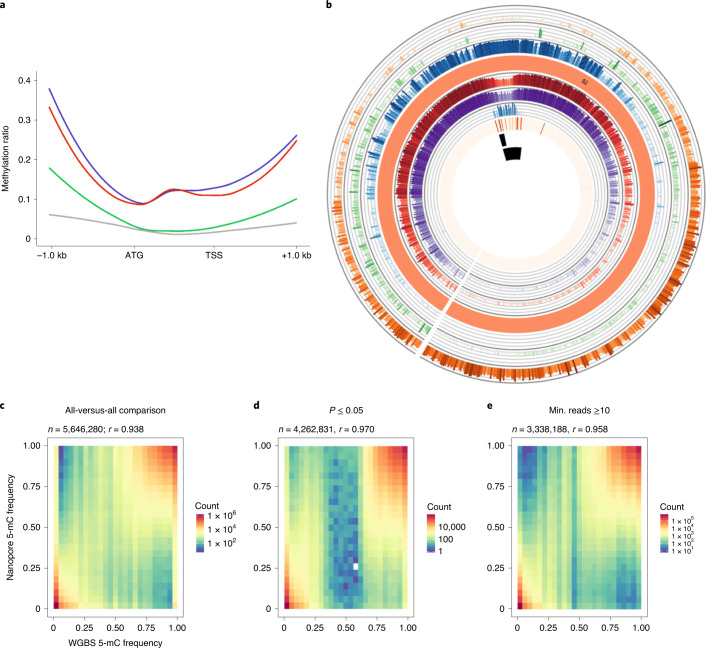
Comparison of methylation data from WGBS and ONT sequencing in Ni100. **a**, Genome-wide WGBS and ONT methylation profile of syntenic genes: CpG (purple), CHG (green), CHH (grey) and CpG by ONT (red). **b**, Genome features of the B2 chromosome of the Ni100-LR assembly, from outer to inner circle: gene density, class II DNA transposons, class I retrotransposons, chromosome cartoon, methylation profile from ONT data, methylation profile based on WGBS, ALE copia, CRB, *B. nigra-*specific centromeric tandem repeat, putative centromere region. This plot was developed using the AccuSyn tool (https://accusyn.usask.ca/). **c**–**e**, Comparison of 5-mC frequency detected by WGBS and ONT; frequency distribution plot without filtering (**c**) and with filtering based on either calls *P* ≤ 0.05 (**d**) or minimum (min.) ONT read depth of 10 (**e**).

Oxford Nanopore Technology allows direct identification of base modifications such as 5-methylcytosine (5-mC)^35^, although this has yet to be demonstrated in plant genomes. Nanopolish was used to detect 5-mC in the CG context in the ONT unassembled reads. The 5-mC calls for Ni100 had an area under the curve score of 0.9, with calls made at 58% of sites using the default threshold of 2.5 for the log-likelihood ratio (Supplementary Fig. 15). The resultant calls were compared with methylation status detected using whole-genome bisulfite sequence (WGBS) data, and showed excellent correlation irrespective of filtering for quality of call (*R* = 0.93–0.97; Fig. 4c–e). A comparison of C2 genome methylation frequencies generated using the two methods showed a slightly lower correlation (0.68–0.80), suggesting that raw read error rate and sequence depth play a crucial role in analysis of methylation using ONT reads (Supplementary Fig. 16). As perhaps expected, the observed methylation showed patterns similar to those detected for related *Brassica* diploids^18^, with a higher prevalence of 5-mC in repeat sequences and lower methylation rates across annotated gene bodies (Fig. 1 and Supplementary Fig. 17). The efficacy of ONT calls in the biological context of gene proximity mirrored the pattern observed for the WGBS data, where methylation increases at the transcriptional start and stop sites (Fig. 4a). Because Nanopolish employs short *k*-mers in its strategy to make a call, this could impact physically linked calls; however, a comparison of the two approaches to identifying differentially methylated CG islands was in agreement, with some variance in the individual site calls within the island (Supplementary Fig. 18).

Of note, there were regions of reduced methylation observed for each chromosome that were also associated with regions of lower gene and higher repeat density. Centromeric regions have been associated in *Brassica* species and, more specifically in *B. nigra*, with particular sequences including centromeric retrotransposon of *Brassica* (CRB) and a B genome-specific short repeat fragment (pBN 35)^7,36^. The distribution of these centromere-associated repeats aligned with the detected hypomethylated regions. Furthermore, members of the more prevalent ALE family, which also has >70% homology with CRB, localized to the same region (Fig. 4b and Supplementary Fig. 19). More recently, sequences identified through interaction with the centromere-specific histone, CENH3, have been sequenced for *B. nigra*, which co-aligned with the hypomethylated regions, suggesting capture of much of the active centromere^37^.

**Fig. 5:**
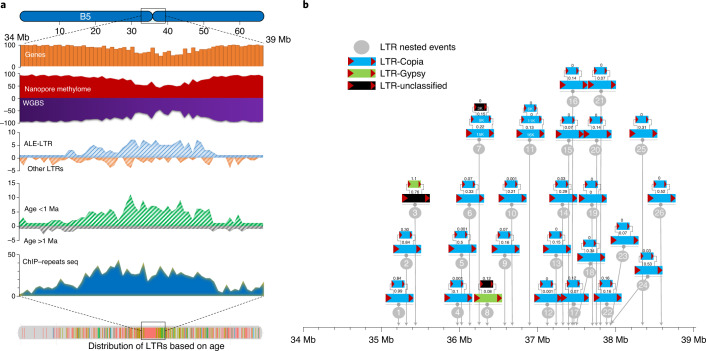
Characterization of centromeric region of chromosome B5 of Ni100-LR genome. **a**, Distribution of various genomic features on the 5-Mb centromere region, including genes, methylome (ONT and WGBS) and full-length LTRs (ALE-LTR and 13 other family LTRs); distribution of young (<1 Ma) and old LTRs (>1 Ma); and distribution of centromeric repeat sequences of *B. nigra* based on chromatin immunoprecipitation (ChIP) analysis of CENH3 (ref. ^38^). **b**, Nested insertion of full-length LTRs in the centromeric region. Age (in Ma) is shown above each element.

Although analyses of nested LTRs have generally been limited to cereal genomes^38^, they would be expected to play a major role in the evolution of chromosome structure and, specifically, in repeat-dense regions such as centromeres. ALE-LTRs were prominent in the centromeric regions and revealed high levels of nested insertion (Fig. 5 and Supplementary Tables 20 and 21). Overall, 262 nested transposable element events were found throughout the Ni100-LR genome of which 68% (179) were in centromeric regions. Across all events, most involved two LTRs while ten events involved more than two LTRs (Fig. 5b and Supplementary Table 21). In-depth characterization of nested TEs in the centromeric region of chromosome B5 revealed that 24/26 of nested transposable element events were created by ALE-LTRs, and all bar one of the events involved the same family member inserting into the host LTR (Fig. 5). The predominantly young age (<1 Ma) of the nested elements suggests continuous and recent rearrangement of the centromeric regions by this mechanism (Fig. 5b).

**Fig. 6:**
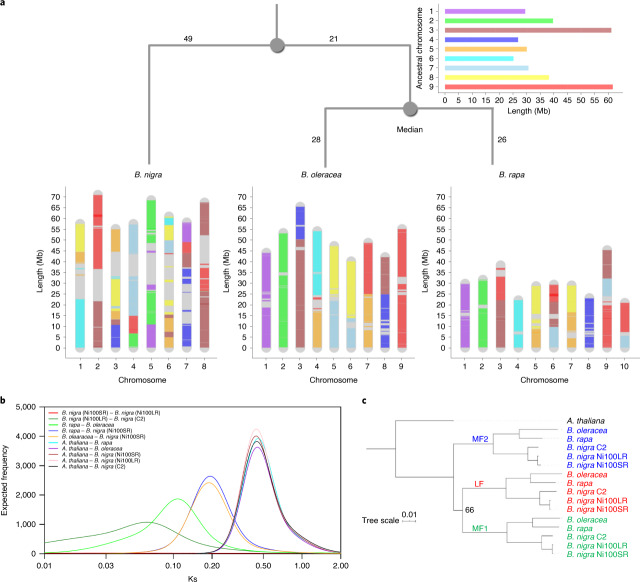
Genome rearrangements and evolution of *Brassica* species. **a**, Development of *B. rapa*, *B. nigra* and *B. oleracea* genomes based on ancestral genome. Blocks are ‘painted’ with colours corresponding to ancestral chromosomes. **b**, Divergence time estimation based on Ks distributions. Gaussian mixture models fitted to frequency distributions of Ks values obtained by comparing pairs of syntelogs between different *Brassica* species or the subgenomes of each species are shown. **c**, Phylogenetic relationship between the subgenomes of different *Brassica* species. A maximum-likelihood tree constructed based on concatenated sequences of 1,150 syntelogs between *A. thaliana* and each of the subgenomes (LF, MF1 and MF2) of *B. rapa*, *B. oleracea* and *B. nigra* is presented. Clade support values near nodes represent bootstrap proportions in percentages. All unmarked nodes have absolute support.

Much effort has been placed on defining an ancestral Crucifer genome that predates the supposed *Brassica-*specific WGT event^39^. Ancestral karyotype blocks were constructed for C2-LR and Ni100-LR based on shared gene content and order for orthologous copies of each *A. thaliana* gene (Supplementary Fig. 20 and Supplementary Table 9). Based on the two-step mode of genome evolution inferred from the genomes of *B. rapa* and *B. oleracea*^40^, which is predicated on genome dominance in newly formed polyploids, as expected the blocks were found predominantly in three copies but with biased genic content. The least fractionated genome maintains approximately 70% of the orthologous gene copies, while the most fractionated, 1 (MF1) and MF2, retain approximately 49 and 42%, respectively (Supplementary Fig. 6a). A phylogenetic analysis of the triplicated orthologues confirmed a shared WGT among the *Brassica*s, with genes from across the three species of each triplicated genome being more similar than those within the same species (Fig. 6c). Some smaller genomic regions were found in additional syntenic blocks in each genome, which could represent more ancient whole-genome duplication events or further localized segmental translocations. These supplementary blocks were more prevalent in the CN115125 genome and could explain the higher prevalence of duplicated genes in this genome (Supplementary Fig. 6c and Supplementary Table 9).

Genomic differences or similarities among species, as well as the mechanisms by which genomes evolve, can be identified by comparing the order in which genes or syntenic blocks appear in both close and distant relatives^41^. The changes to block orders are defined in terms of certain rearrangement operations within a chromosome or between chromosomes, such as reversal, transposition, fusion, fission and translocations. These types of operation can be abstracted computationally as a series that results in a change in the linear ordering of genes, which can then be used to calculate the ‘genomic distance’ between one version of a genome and another based on the most parsimonious evolutionary process. The double-cut-and-join (DCJ) model^42^ was used to calculate pairwise genomic distances between the three *Brassica* diploid genomes: *d*_*rapa,nigra*_ = 96; *d*_*nigra,oleracea*_ = 98; *d*_*rapa,oleracea*_ = 52. In addition to measurement of genomic difference or similarity, the order of blocks in extant genomes provides rich information that can be used in reconstruction of ancestral gene orders.

The ancestral *Brassica* genome, which minimizes the number of rearrangements and thus genomic distances between the three genomes, has nine ancestral chromosomes with a genome size of 321 Mb (Supplementary Fig. 21 and Supplementary Tables 22 and 23), consisting of 178 blocks. Each block in the ancestral genome was mapped to the three extant genomes, as shown in Fig. 6a, while Supplementary Fig. 22 shows the detailed position of each ancestral block and its relative orientation. Based on calculated genomic distances between the ancestral genome and each diploid, a rooted ultrametric phylogenetic tree was approximated (Fig. 6a) where the position of the ancestor minimizes the total genomic distance. Based on the molecular clock hypothesis, which assumes a constant rate of change within lineages, the ancestor would be the most recent common ancestor of *B. rapa* and *B. oleracea* while an overall ancestor would be inferred almost 1/3 of the way along a path from the median to *B. nigra*. The genomic distance between the genomes corresponded with the age of divergence estimated from the synonymous substitutions (Ks) rates among the coding regions of orthologous gene pairs across the genomes, with *B. oleracea*/*B. rapa* having diverged from *B. nigra* some 11.5 Ma while they diverged from each other only 6.8 Ma (Fig. 6b and Supplementary Table 24).

## Discussion

Recent advancements and cost reductions in LR sequencing technologies are facilitating the generation of high-quality genome assemblies, even for species that have evolved through recursive whole-genome duplication (WGD) events^43^. High-quality and highly contiguous assemblies were generated for two genotypes of the mesopolyploid *B. nigra* using nanopore sequencing, chromosome-level scaffolding with Hi-C and genetic mapping data. Remarkably, the final contig N50 length was 17.1 Mb (Ni100-LR), one of the longest among the 324 plant genomes published to date (Supplementary Fig. 23 and Supplementary Table 25). Comparing the two ONT assemblies, the Ni100-LR assembly was better in terms of contiguity and capture of repeat-rich centromeric regions, reflecting rapid improvements in the technology and suggesting the importance of both read length (11 versus 20 kb) and read coverage (29 versus 64×). Accurate quantification of errors in the Ni100 nanopore assembly, by comparison with an Illumina SR assembly of the same genotype, suggested an accuracy of 99.986%, which was improved only marginally (99.998%) with eight rounds of SR polishing, suggesting that nanopore reads can provide highly accurate assemblies of complex genomes. The error rate was higher for the CN115125 assembly (0.8 versus 0.2%), again reflecting recent improvements in ONT technology. The determined error rate may also be impacted by genome complexity, since matching of Illumina reads to regions of low complexity is generally limiting, and thus it might be expected that error rates will be higher in such regions.

The recognition that both small (copy number, presence/absence) and large (chromosomal rearrangements) SVs play an important role in controlling key agronomic traits is gaining traction^44^, yet deciphering such variation with SR data has proved problematic^45^. Although long-read sequencing technologies have distinct advantages in predicting SVs^46^, the current limitation is in regard to developing and training software to accurately identify such variants. The current analyses provided a detailed picture of large-scale rearrangements that differentiate genotypes, and also used two widely accepted software tools for detection of SVs, and cross-validation was attempted to improve the accuracy of the calls. The large difference in the number of events discovered by the different protocols probably reflects a combination of a higher false and a lower positive discovery rate between the two. Considering only the cross-validated calls, a large number of events differentiated the two *B. nigra* genotypes, many of which would impact gene expression and potentially phenotype, thus underlying the need for improved tools for SV analyses to capture this valuable information.

It is well established that LR sequence data provide a more comprehensive coverage of the genome^47^, perhaps most obviously reflected in the increased capture of low-complexity repeat sequences. Repeat analysis revealed about 14% more repeats in the LR assembly of Ni100 compared to the SR assembly (54 versus 41%) and, in particular, a more complete assembly of the repeat-rich centromeric and pericentromeric space. Centromeres are structures essential for the maintenance of karyotype integrity during meiosis, ensuring the fertility of developed gametes through strict inheritance of full chromosome complements; nevertheless, centromeres still remain under-explored, especially in larger genomes. Although the active centromere is incredibly diverse in size and sequence among species, it is characterized through its cohesion with the centromere-specific histone H3-like protein, CENH3, and it has been suggested that association with CENH3 is controlled through epigenetic means, including a decrease in CG methylation^48^. Direct CG methylation profiling using the ONT data not only suggested the efficacy of this approach (93–97% correlation with WGBS) but also demarcated the active centromere in the assembly, with hypomethylated regions being co-located with known and new centromeric repeat sequences. At least three of the chromosomes for Ni100 (B1, B3 and B8) showed multiple hypomethylated islands within or adjacent to the putative centromere region, which also coincided with centromeric-specific repeats (Supplementary Fig. 24). It was noted for *B. rapa* that such repeats found outside the presumed centromeric region may represent evidence of ancient palaeo-centromeres, remnants of WGD events^21^. However, all additional sites coincided with hypomethylation, suggesting functionality of the regions. This could imply potential scaffolding errors remaining in the dense repeat regions although, interestingly, even though the data were more limiting the same pattern appeared to be apparent for the CN115125 genotype, which could suggest a dispersed structure for the active centromeric region^8^. Where comparison was feasible, the two genotypes showed a common dichotomy of centromeric regions, with conservation of gene content but rapid divergence in sequence constitution driven by changes in retrotransposon composition.

Recent work in *B. nigra* to uncover centromere-specific sequences through their association with CENH3 indicated that, unlike its diploid relatives and almost all analysed plant genomes, *B. nigra* contains no tandemly repeated satellite DNA^6,37^. Similarly, although no characteristic tandem repeat was found in the LR assemblies, analyses of assembled full-length LTRs revealed recently amplified (< 1 Ma) elements, in particular ALE-LTRs in the Ni100-LR genome (Fig. 5). Notably all eight of the centromeric regions displayed a very similar structure with a core region largely populated with ALE elements flanked by dense islands of the previously described pBN 35 short repetitive element (Supplementary Fig. 11 and Supplementary Table 20). Interestingly a number of the retro-elements encompassed the pBN 35 sequence within their LTR domains, which might suggest its capture during element activity. Rapid amplification of young LTRs in a nested insertion fashion was observed in all Ni100 centromeric regions (Supplementary Table 21), a phenomenon that was not obvious for the available LR assembly of *B. rapa* (Supplementary Table 26)^21^. Nested TE insertion is a prevalent phenomenon among monocots, but has been identified only infrequently among dicots^49^. The detected recent nested insertion events involving a single family suggest that ALE or related LTRs might play an important role in the rapid divergence of centromeres in *B. nigra*, similar to that found when comparing the centromeric region of two rice genotypes^50^. It was postulated that retrotransposons are actively recruited to the functional centromere; >90% of the *B. nigra* sequences found to be associated with CENH3 showed significant homology to ALE elements, providing circumstantial evidence for the role of CENH3 in their accumulation at the centromere core (Supplementary Table 27). Further studies are required to fully establish the role of these elements in centromere function in *B. nigra* and, indeed, the LR assembly resources developed for the Ni100 genotype could be leveraged as a model for centromere function research in future.

Although an ancestral block structure was established for *Brassica*s some years ago, it was largely defined manually and there has been no clear resolution of the events separating genomes. Syntenic relationships between species have been instrumental in gene discovery and in dissecting genome evolution that can impact technology transfer across species. The improved assemblies for all three diploid *Brassica* genomes allowed an ancestral *Brassica* genome (*n* = 9) to be resolved based on 178 syntenic blocks. The calculated genomic distance between the genomes reflects the age of divergence between the B and A/C genome lineages (Fig. 6a). While *B. rapa* and *B. oleracea* have chromosomes sharing extensive homology with ancestral chromosomes, the extent of the rearrangements separating the B genome would explain the limited genic exchange that has been possible across the two lineages. Therefore, capturing new diversity from the third *Brassica* genome for crop improvement strategies in its related species may be more efficient using next-generation breeding techniques such as clustered regularly interspaced short palindromic repeat/Cas9. The defined block relationships between the genomes also provide further avenues for studying centromere evolution because, among the 27 centromeric regions across the three species, 26 had adjacent or flanking conserved blocks found across either two or three of the genomes, suggesting evolutionary conserved positions (Supplementary Fig. 22; compare A8, C8 and B7).

The ability to generate, relatively quickly and affordably, contiguous genome assemblies provides a platform for the development of true pan-genomes for many species. Such assemblies will allow an accurate comparison of not only gene content, but also repeat composition and distribution, and reveal the range and complexity of structural variation. There are still some limitations, and assemblies of neopolyploid genomes will need to be assessed to determine whether the technology can routinely differentiate young WGD events. However, with continuing improvements to the technology and optimization of software dedicated to analyses of these new data types, resolution of these problems should be swift.

## Methods

### Plant materials and DNA extraction

*Brassica nigra* CN115125 (C2) and Ni100 were grown in a greenhouse at Agriculture and Agri-Food Canada, Saskatoon Research and Development Centre, under 20/18 °C, 16/8-h days. Leaf tissue was collected from 3-week-old plants after 2 d of dark treatment, flash-frozen and stored at −70 °C. Nuclear isolation was performed as described in ref. ^51^, and high-molecular-weight DNA was extracted using a modified cetyltrimethylammonium bromide (CTAB) method^52^. Briefly, approximately 20 g of leaf tissue was homogenized in 200 ml of ice-cold 1× Hanks’ buffered salt (HBS) solution (0.01 M Trizma base, 0.08 M KCL, 1 mM spermidine, 0.01 M EDTA, 0.5 M sucrose, 1 mM spermine plus 0.15% β-mercaptoethanol). Five millilitres of 1× HBS plus 20% Triton X-100 was added to the homogenate and mixed slowly with a magnetic stir bar for 1 h on ice, then filtered through two layers of cheesecloth and one layer of Miracloth. The nuclei were pelleted by centrifugation of homogenate at 1,800*g* for 20 min at 4 °C. The pellet was washed by resuspension in 1× HBS plus 0.5% Triton ×100 on ice and centrifuged three times at 1,800*g* for 20 min at 4 °C to purify the nuclei. The final pelleted nuclei were resuspended in 10 ml of lysis buffer (100 mM TrisHCl, 100 mM NaCl, 50 mM EDTA, 2% CTAB) treated with proteinase K, followed by RNAase A (37 °C for 30 min), and high-molecular-weight DNA was extracted after two cycles of phenol/chloroform clean-up and ethanol precipitation. DNA quality and quantity were measured using an Agilent Bioanalyzer and Qubit fluorometer, respectively.

### ONT sequencing and reads processing

The C2 genome was sequenced on a MinION while the Ni100 genome was sequenced on a GridION. For the C2 genome, 1D (SQK-LSK108) and 1D^2^ (SQK-LSK308) genomic DNA libraries were prepared following the nanopore protocol (https://community.nanoporetech.com/protocols). For size-selected DNA, 4 µg of DNA was sheared with a Covaris g-TUBE to obtain 10-kb fragments. Two micrograms of sheared and un-sheared DNA was used for library preparation for both the 1D and 1D^2^ methods. For the Ni100 genome, 1D (SQK-LSK109) and Rapid (SQK-RAD004) libraries were prepared for sequencing on GridION. MinION sequencing used MinKnow v.1.4.2 with albacore (v.1.1.2) live base calling, enabled with default parameters. ONT reads with read quality score ≥10 (q10) were filtered from the ONT fastq files (Supplementary Table 3). For the Ni100 genome, sequenced using GridION, MinKnow 2.0 and live base calling was completed with Guppy, and ONT reads with read quality score ≥7 (q7) were used for assembly. Nanostat^53^ was used to compute the sequencing statistics for each run with both raw and quality-filtered data.

### Illumina sequencing

Genomic DNA extracted as above was used for whole-genome Illumina sequencing. For CN115125 (C2), 2 µg of DNA was fragmented using a Covaris sonicator to obtain 350-bp fragments, and a TruSeq DNA PCR-Free library was prepared following the manufacturer’s protocol (Illumina, Inc.). The normalized library was paired-end sequenced in 2 × 101 bp and 2 × 250 bp rapid-run mode on the HiSeq 2500 platform (Illumina, Inc.). In total, >82 Gb of SR sequences with ~137× physical coverage were generated for C2 (Supplementary Table 28). For Ni100, whole-genome shotgun Illumina paired-end (300–700 bp insert size) and Illumina and Roche/454 (Life Sciences) mate-pair libraries (3–45 kb insert size) were developed following the manufacturers’ protocols. In total 115 Gb (~192× physical coverage) were sequenced and used for whole-genome assembly by SOAPdenovo (v.1.05) following a previously documented approach^18^ (Table 1 and Supplementary Table 28).

Total RNA was extracted from bud, flower, leaf, seedling, root and silique tissue samples for Ni100, and from leaf and bud samples for C2, using the RNeasy plant mini kit (QIAGEN), including on-column DNase digestion (Supplementary Table 28). Total RNA integrity and quantity were assessed on a Bioanalyzer (Agilent). Illumina TruSeq RNA-sequencing (RNA-seq) libraries were prepared, and 125-bp, paired-end sequencing was performed using the Illumina HiSeq 2000 platform. A total of 11 and 39 Gb raw Illumina RNA-seq data were generated for C2 and Ni100, respectively (Supplementary Table 28). Reads were filtered for low quality (<q30), adaptor sequence, potential PCR duplicates and length (<55 bp) with Trimmomatic (v.0.32). RSEM^54^ (rsem-calculate-expression) was used to calculate expression, in transcripts per million.

### Genome size estimation based on *k*-mer analysis

Jellyfish v.2.2.6 was used to estimate *k*-mer frequency distribution based on the subset (~35 Gb) of raw 2 × 250 PE Illumina reads with a *k*-mer length of 17. The output histogram was uploaded to findGSE to estimate genome size, heterozygosity and repeat fraction^55^. Analysis has shown that genome size is about 570 and 607.8 Mb for Ni100 and C2, respectively, and was used as a haploid genome size for the study (Supplementary Fig. 25).

### Nanopore sequence assembly and polishing

Raw ONT fastq data were filtered for quality at q10 and q7 for C2 and Ni100, respectively, and the resulting reads were error corrected using CANU 1.6 with default parameters^24^. The C2 filtered data were assembled with three different assemblers (SMARTDenovo, wtdbg, Miniasm). Minimap2 was used to generate overlaps of corrected reads, with *k* = 24 and other default parameters (-csw5 -L100 -m0) followed by assembly using miniasm^24,56^. SMARTDenovo (https://github.com/ruanjue/smartdenovo) was used with *k* = 24 and recommended parameters. The wtdbg tool (https://github.com/ruanjue/wtdbg) was used to assemble the reads, with *k* = 17, *k* = 24 and default parameters (-H -S 1.02 -e 3). The best assembly for the C2 genome (S4), based on contiguity and genome coverage, was selected for further analysis (Supplementary Table 1). Based on this preliminary analysis, the Ni100 genome were assembled using SMARTDenovo with *k*-mer 24 and default parameters. Both draft assemblies were polished using eight iterations of PILON^25^ with available Illumina reads.

### Contig scaffolding

Leaf tissue from C2 was provided to Dovetail genomics (Santa Cruz), who prepared and sequenced CHiCAGO and Hi-C libraries. The polished assemblies, CHiCAGO and Dovetail Hi-C library reads were used as input for scaffolding using Dovetail’s HiRise pipeline^57^. A modified SNAP read mapper uses CHiCAGO and Hi-C reads to align to the draft assembly, while HiRise produces a likelihood model for the genomic distance between read pairs, computing the optimum threshold to join contigs and identify putative misjoins.

A genetic map derived from genotyping-by-sequencing data of a back-cross population of 72 *B. nigra* lines, derived from the Ni100 × double-haploid line A1//Ni100, was used to anchor contigs from all assemblies to the pseudo-molecules. In total, 20,689, 19,666 and 21,034 loci were anchored to the genome assemblies of C2, Ni100-SR and Ni100-LR, respectively. The assembly was confirmed using genome-ordered graphical genotypes (GOGGs)^58^ based on transcriptome re-sequencing of lines from the *Brassica juncea* VHDH mapping population^59^ and genome re-sequencing of lines from the *B. juncea* YWDH population^60^. GOGGs also enabled the incorporation of four previously unanchored scaffolds into the chromosome assemblies. Sequences of restriction fragment length polymorphism clones used to generate the genetic map in ref. ^61^ were aligned to the assemblies to name and orient the pseudo-molecules accordingly, based on the internationally agreed standard (http://www.Brassica.info). A look-up table comparing chromosome (linkage group) names between the two published nomenclatures for the B genome is shown in Supplementary Table 6.

### Assembly quality assessment

Quality of the assembly was estimated using single-copy orthologous gene analysis (BUSCO v.0.2)^26^ with Embryophyta OrthoDB v.9. The 1,440 genes were searched in the assembly using Augustus (v.3.2.1)^62^, NCBI’s BLAST (v.2.2.31+)^63^ and HMMER (v.3.1b2) by BUSCO. In addition, genome discrepancies were estimated using qualimap^27^ by mapping Illumina reads against the polished assembly. Bowtie-2 (ref. ^64^) with default parameters was used for mapping of Illumina reads against the assembly.

### Genome annotation

RNA-sequencing (39 Gb) data for Ni100 and C2 were aligned against their respective genome assemblies using STAR v.2.7 (maximum 3% mismatches over 95% read length), and subsequently assembled using the Trinity (v.2.8.4) genome-guided approach with default parameters. In total, 110,767 and 124,851 transcripts were assembled for Ni100 and C2, respectively. The assembled transcripts, along with protein sequences from *A. thaliana*, *Arabidopsis lyrata*, *B. rapa* and *B. oleracea*, were used as evidence for the MAKER-P annotation pipeline^65^. Snap and Augustus ab initio predictors were configured for use by MAKER-P in hint-based mode, using protein and transcript as input evidence. Approximately 6% of the predicted gene models were found to be misjoined based on *A. thaliana* gene structure and *B. nigra* transcript evidence, and were split into two or more alternate models. PASA (v.2.3.3) software^66^ was then used to further assemble Trinity output and to incorporate the transcript alignment evidence into MAKER gene annotation. In total, 59,877 and 67,030 coding genes were annotated for Ni100 and C2, respectively. Of the annotated genes, 48,621 (81.2%) of Ni100 and 54,586 (81.4%) of C2 gene models have expression values of transcripts per million (TPM) > 0. BLASTP revealed that 55,022 (92.0%) of Ni100 and 59,780 (89.2%) of C2 genes have significant hits (cut-off 10 × 10^–5^) against the Uniprot plant database.

The gene-naming convention proposed for *B. rapa* v.3 (ref. ^21^) was used with minor modifications: Bni (for *B. nigra*) followed by the chromosome number with leading zero, and the letter ‘g’ (for gene)—for example, B01g (for B genome chromosome 1). Six-digit gene numbers were assigned in steps of ten, with leading zeros from top to bottom of chromosomes. Following the gene number and separated by a period, to distinguish genome versions and between genotypes, ‘2N’ was assigned to Ni100 LR (genome version 2) and ‘1C2’ to C2 (genome version 1)—for example, BniB01g023500.2N. Low-confidence genes were defined as those models with neither transcriptome evidence support nor significant hits to the Uniprot plant database. Low-confidence genes were named similarly as described above but with the letter ‘p’ to distinguish them.

### Repeat annotation

A de novo repeat library was developed using RepeatModeler (v.1.0.11; http://www.repeatmasker.org/RepeatModeler/), which uses two de novo repeat-finding programmes (RECON and RepeatScout) for identification of repeat families. After removal of potential false positives based on homology with *A. thaliana* gene models, a total of 374 repeat models were retained. In addition, a previously developed repeat library for *B. nigra* Ni100, which contains 950 repetitive elements, was merged to develop a final repeat library with 1,324 elements that was used for repeat annotation in the whole genome. Repeatmasker was employed to estimate repeat copies, proportion and distribution into the genome^67^.

Centromeric location was identified based on the distribution of centromere-associated repeats such as CRB, *B. nigra-*specific centromere-associated repeat (pBN35—X16588.1) and CENH3-associated sequences^36,68^. Based on the distribution of these elements using BLAST, centromere regions were located in the assembly.

Full-length, long terminal repeat retrotransposons were identified from both genome assemblies using LTR_harvest^69^ and LTR_Finder^70^. The resulting outputs (.scn) were fed into the LTR retriever programme^71^ to extract FL-LTR-RTs. Copy number, distribution and divergence time of LTR-RTs were comparatively analysed between the two reference genomes. FL-LTR-RTs were classified into different families based on homology with the repeat library from Repeat Explorer^72^. Potentially conserved FL-LTR-RTs were identified through reciprocal BLAST analyses of the unique flanking DNA sequences, when paired flanking DNA was positioned syntenically and within 5 Mb of the position in the alternate genome they were considered conserved. The complexity of the *B. nigra* centromere regions was represented by a graphical network formed from an adjacency matrix of FL-LTR-RTs (Supplementary Fig. 19b,c). The matrix was determined from sequential pairing of annotated TE elements present in the centromeric or pericentromeric definitions. All analyses were performed using R, and the graphical representation made using the igraph package.

Phylogenetic analysis was done using the reverse-transcriptase (RT) domain sequences of ALE and OTA elements from the three *B. nigra* genomes. RT domains obtained from the Pfam database (accession nos. PF07727 and PF00078) were used as a query to search against the FL-LTRs of ALE and OTA sequences, respectively, by BLASTx, and the best hit with a minimum of 200-bp overlap with query sequences was used for further analysis. RT domain sequences of ALE and OTA families from the three *B. nigra* genomes were aligned separately by the clustalW aligner, and a tree was generated using the neighbour-joining method with 500 bootstrap replications by MEGA7. FL-LTR-RTs annotated for the whole genome using the LTR retriever were manually analysed to identify nested TE insertion.

### Gene and genome evolution

OrthoFinder v.2.2.7 (ref. ^31^) was used to identify members of gene families and assess their expansion in C2 and Ni100-LR, by clustering annotated genes with the closely related species *B. rapa*^21^, *B. oleracea*^18^ and *A. thaliana* (Araport11) (Supplementary Table 11). CAFE v.4.2.1 (ref. ^32^) was used to identify expansion, contraction and rapidly evolving gene families among the six genomes based on the orthogroups obtained from OrthoFinder analysis.

Synteny analysis was performed to identify syntenic genes between *B. nigra* Ni100-LR/C2 and *A. thaliana* using the *A. thaliana* proteome (Araport10) as described previously^18^. Briefly, based on best BLASTP values (1 × 10^–20^ or better), syntenic gene pairs between *B. nigra* and *A. thaliana* were employed in DAGChainer with default parameters to compute the chain score^73^. Manual curation based on better chain score was done to create the final syntelog table (Supplementary Table 22). Tandemly duplicated and proximal genes were identified following an approach previously reported^74^. Briefly, potential homologous pairs between each of three genomes were identified by all-versus-all BLASTP at 1 × 10^–10^. MCScanX (default parameters) was then used to identify duplicated pairs from Ni100 and C2 that formed intra-species syntenic chains. These pairs were set aside and classified as WGD-derived gene pairs. The remaining pairs (or BLASTP hits) were classified as either tandem (adjacent to each other on the same chromosome) or proximal (>1 and ≤10 genes on the same chromosome).

For phylogenetic analysis, a data matrix consisting of 1,150 syntelogs retained in *A. thaliana* and each of the subgenomes (least fractionated, LF; most fractionated 1, MF1; most fractionated 2, MF2) of *B. rapa*, *B. oleracea* and *B. nigra* was constructed. Sequences from individual syntelog gene sets were aligned using ClustalW v.2.1 (ref. ^75^), and poorly aligned regions were removed using trimAL v.1.2 (ref. ^76^). Trimmed sequences were concatenated using the Phyutility programme^77^ to produce the final data matrix comprising a total alignment length of 807,943 bp. Phylogenetic relationships were inferred using the maximum-likelihood method implemented in RAxML v.8.2.12 (ref. ^78^), using rapid bootstrapping (100 replications) and a GTRGAMMA substitution model. The resulting phylogenetic tree was visualized using the interactive Tree of Life v.4 web server^79^.

In the DCJ model^42^, two genomes being compared are represented as a ‘breakpoint’ graph allowing calculation of the genomic distance and DCJ distance between genomes. For ancestral genome reconstruction, given gene orders in a set of genomes *G* and a distance measure *d*, the median problem is to find an ancestral genome (*m*) that minimizes the sum of distances: $d^{\mathrm{\Sigma }} = d({{m,{{g}})}},\forall{{{g}} \in G}$. The median problem is known to be NP-hard under the DCJ distance^80^. Given three genomes and *d*_i,j_ as the pairwise DCJ distance between genomes i and j, the metric $d^{\mathrm{\Sigma }}$ has the following properties—the lower bound is $d_{\mathrm{l}}^{\mathrm{\Sigma }} = \frac{{d_{1,2} + d_{2,3} + d_{1,3}}}{2}$ and the upper bound is $d_{\mathrm{u}}^{\mathrm{\Sigma }} = d_{1,2} + d_{2,3} + d_{1,3} - {\mathrm{max}}\{ d_{1,2},d_{2,3},d_{1,3}\}$^81^. Therefore, given the pairwise DCJ distances calculated for the three genomes, *B. rapa*, *B. nigra* and *B. oleracea*, $d_{\mathrm{l}}^{\mathrm{\Sigma }} = 123$ and $d_{\mathrm{u}}^{\mathrm{\Sigma }} = 148$. The ASMedian-linear algorithm^81^ is designed to find exact solutions to the DCJ median problem on multi-chromosomal genomes. It uses a divide-and-conquer approach to decompose the multiple breakpoint graph to its ‘adequate’ subgraphs, find optimal solutions for its parts and then combine the optimal solutions. A total of 25,866 orthologous genes were identified between the genomes of *B. rapa*, *B. nigra* and *B. oleracea.* These genes were used to identify 178 unique syntenic blocks where, although distances between blocks were estimated based on fractionated genes, block reversals were used to fix block breakpoints. Finally, an ancestral genome was calculated using the ASMedian-linear algorithm as nine ancestral chromosomes with a genome size of 321 Mb. The calculated ancestral genome (*m*) under the DCJ model generates a minimum total DCJ distance: ${\it{d}}^{\mathrm{\Sigma }} = 124$, where *d*_*m,*__rapa_ = 26, *d*_*m,*__nigra_ = 70 and *d*_*m,*__oleracea_ = 28.

Distribution of synonymous substitutions (Ks) was performed as described previously^14^. Briefly, for each pair of syntelogs between the *Brassica* species or the subgenomes of each *Brassica* species, protein sequences were aligned using ClustalW v.2.1 (ref. ^7^) and the corresponding codon alignments were produced using PAL2NAL^82^. Ks values for each sequence pair were calculated using the maximum-likelihood method implemented in codeml of the PAML package^83^ under the F3x4 model^84^. Histograms were generated using log-transformed Ks > 0.001. Gaussian mixture models were fitted to the ln(Ks) values using the R package Mclust, and the number of Gaussian components, the mean of each component and fractions of data were calculated. The Bayesian information criterion was used to determine the best-fitting model to the data. The fit of the determined models was confirmed by *χ*^2^ tests.

The presence of resistance genes was identified using the RGAugury pipeline (v.2017.10.21)^85^; transcription factors, transcription regulators and protein-kinase families were identified by iTAK (current v.1.7, 13 May 2016)^86^.

### Structural variant analysis

Structural variants such as insertions, deletions, inversions, duplications and translocations were identified using both Ni100-LR and C2-LR assemblies. Raw long reads of both genomes were mapped using NGMLR LR aligner on Ni100-LR as a reference, and SVs were called using Sniffles with a minimum read depth of 20 (ref. ^33^). Likewise, SVs were predicted using C2-LR as a reference assembly. Furthermore, cross-validation of SVs identified by Sniffles was done using another SV identifier, Picky, with the same read depth of 20 (ref. ^34^). SVs shared by both callers were identified as high-quality SVs and used for further analysis. At least 15 random SVs of each type were assayed manually, and suggested almost 100% prediction accuracy for deletions and insertions; however, some of the larger predicted events appeared less reliable and it was apparent that a number of deletions had been overlooked, suggesting that the criteria may have been too stringent. Seven SVs caused by repeats were manually validated by gel analysis (Supplementary Table 29).

### WGBS

Genomic DNA was isolated from leaf tissue of *B. nigra* Ni100 with two biological replications, and from leaf and bud tissue from *B. nigra* CN115125 nuclei, using QIAGEN’s DNeasy Plant Kit following the manufacturer’s protocol. A Zymo Research EZ DNA Methylation kit was used for bisulfite conversion on 100 ng of DNA, along with 0.5% w/w unmethylated lambda DNA (Promega), included to evaluate bisulfite conversion efficiency. Library construction was performed according to the Illumina TruSeq DNA Methylation Kit Reference Guide (no. 15066014, v.01). The libraries were quantified as above and paired-end sequenced (2 × 125 bp) using an Illumina HiSeq 2000.

Quality-filtered WGBS reads were used to analyse cytosine methylation ratios following alignment using BSMAP (v.2.9) (Supplementary Table 28)^87^. Lambda DNA was included in each library as a control to estimate bisulfite conversion efficiency. In all instances the conversion rate was estimated at >99%. The evidence to assign the methylatation status of each cytosine surveyed was determined using binomial probability distribution. Methylation patterns were determined and summarized using the support from available genome annotation. Methylation patterns were partitioned by context (CG, CHH, CHG), reflecting the underlying biochemistry underpinning their maintenance. Statistical relationships and data organization were performed using custom Perl and R scripts, with support from Datatable, dplyr, stringr, genomation and MethylKit; all graphical summaries were developed using ggplot2. CpG islands were identified by EMBOSS using the cpgplot tool with default parameters^88^. These features were filtered based on position to include only those residing in the 5′ regulator region (−2,000 to 0 bp from ATG) of the annotated gene features. DNA methylation detected using the ONT and WGBS methods was compared at each cytosine occurring in the CG context throughout the filtered islands. Agreement between methylation base calls was assessed at individual loci, and the similarity represented graphically following dimension reduction (Supplementary Fig. 18).

### CpG context in nanopore reads by Nanopolish

Because nanopore reads have the facility to output signals for both methylated and unmethylated cytosine bases, Nanopolish was used to detect the CpG context in the whole genome of Ni100 (ref. ^35^). Nanopolish v.0.10.1 was used to call bases for both methylated and unmethylated bases from raw nanopore reads, and results were filtered as described based on either log-likelihood ratio or read depth.

### Reporting Summary

Further information on research design is available in the Nature Research Reporting Summary linked to this article.

## Supplementary information

Supplementary Information Supplementary Figs. 1–25.

Reporting Summary

Supplementary Table 1. Supplementary Tables 1–29.

## Data Availability

All genome assembly and annotation-associated DNA-seq, RNA-seq and WGBS-seq data have been deposited with NCBI under BioProject ID PRJNA516907. The assembled pseudo-molecules and associated annotation files, along with a Jbrowse instance for each genome, can be accessed at http://cruciferseq.ca. All supporting data are included in the Supplementary Information. Source data are provided with this paper.

## Source data

Source Data Fig. 3. Source data.

## References

[1] Bevan MW (2017). Genomic innovation for crop improvement. Nature.

[2] Abberton M (2016). Global agricultural intensification during climate change: a role for genomics. Plant Biotechnol. J..

[3] Scheben A, Wolter F, Batley J, Puchta H, Edwards D (2017). Towards CRISPR/Cas crops—bringing together genomics and genome editing. N. Phytol..

[4] Michael TP (2014). Plant genome size variation: bloating and purging DNA. Brief. Funct. Genomics.

[5] Lim KB (2007). Characterization of the centromere and peri‐centromere retrotransposons in *Brassica rapa* and their distribution in related *Brassica* species. Plant J..

[6] Lan T (2017). Long-read sequencing uncovers the adaptive topography of a carnivorous plant genome. Proc. Natl Acad. Sci. USA.

[7] Koo DH (2011). Rapid divergence of repetitive DNAs in *Brassica* relatives. Genomics.

[8] Muller, H., Gil, J. Jr & Drinnenberg, I. A. J. The impact of centromeres on spatial genome architecture. *Trends Genet.* **35**, 565–578 (2019). 10.1016/j.tig.2019.05.003 31200946

[9] Sedlazeck FJ, Lee H, Darby CA, Schatz MC (2018). Piercing the dark matter: bioinformatics of long-range sequencing and mapping. Nat. Rev. Genet..

[10] Jiao W-B, Schneeberger K (2017). The impact of third generation genomic technologies on plant genome assembly. Curr. Opin. Plant Biol..

[11] Koren S, Phillippy AM (2015). One chromosome, one contig: complete microbial genomes from long-read sequencing and assembly. Curr. Opin. Microbiol..

[12] Jiao Y (2017). Improved maize reference genome with single-molecule technologies. Nature.

[13] Deamer D, Akeson M, Branton D (2016). Three decades of nanopore sequencing. Nat. Biotechnol..

[14] Kagale S (2014). Polyploid evolution of the Brassicaceae during the Cenozoic era. Plant Cell.

[15] Lysak MA, Koch MA, Pecinka A, Schubert I (2005). Chromosome triplication found across the tribe Brassiceae. Genome Res..

[16] Nagaharu U, Nagaharu N (1935). Genome analysis in *Brassica* with special reference to the experimental formation of *B. napus* and peculiar mode of fertilization. Jpn. J. Bot..

[17] Truco MJ, Quiros CF (1994). Structure and organization of the B genome based on a linkage map in *Brassica nigra*. Theor. Appl. Genet..

[18] Parkin IA (2014). Transcriptome and methylome profiling reveals relics of genome dominance in the mesopolyploid *Brassica oleracea*. Genome Biol..

[19] Liu S (2014). The *Brassica oleracea* genome reveals the asymmetrical evolution of polyploid genomes. Nat. Commun..

[20] Chalhoub B (2014). Early allopolyploid evolution in the post-Neolithic *Brassica napus* oilseed genome. Science.

[21] Zhang L (2018). Improved *Brassica rapa* reference genome by single-molecule sequencing and chromosome conformation capture technologies. Hortic. Res..

[22] Yang J (2016). The genome sequence of allopolyploid *Brassica juncea* and analysis of differential homoeolog gene expression influencing selection. Nat. Genet..

[23] Belser C (2018). Chromosome-scale assemblies of plant genomes using nanopore long reads and optical maps. Nat. Plants.

[24] Koren S (2017). CANU: scalable and accurate long-read assembly via adaptive *k*-mer weighting and repeat separation. Genome Res..

[25] Walker BJ (2014). Pilon: an integrated tool for comprehensive microbial variant detection and genome assembly improvement. PLoS ONE.

[26] Simão FA, Waterhouse RM, Ioannidis P, Kriventseva EV, Zdobnov EM (2015). BUSCO: assessing genome assembly and annotation completeness with single-copy orthologs. Bioinformatics.

[27] Okonechnikov K, Conesa A, García-Alcalde F (2015). Qualimap 2: advanced multi-sample quality control for high-throughput sequencing data. Bioinformatics.

[28] Golicz AA (2016). The pangenome of an agronomically important crop plant *Brassica oleracea*. Nat. Commun..

[29] Wu TD, Watanabe CKJB (2005). GMAP: a genomic mapping and alignment program for mRNA and EST sequences. Bioinformatics.

[30] Bachmann JA, Tedder A, Laenen B, Steige KA, Slotte TJ (2018). Targeted long-read sequencing of a locus under long-term balancing selection in *Capsella*. G3 (Bethesda).

[31] Emms DM, Kelly SJ (2015). OrthoFinder: solving fundamental biases in whole genome comparisons dramatically improves orthogroup inference accuracy. Genome Biol..

[32] Han MV, Thomas GW, Lugo-Martinez J, Hahn MWJ (2013). Estimating gene gain and loss rates in the presence of error in genome assembly and annotation using CAFE 3. Mol. Biol. Evol..

[33] Sedlazeck FJ (2018). Accurate detection of complex structural variations using single-molecule sequencing. Nat. Methods.

[34] Gong L (2018). Picky comprehensively detects high-resolution structural variants in nanopore long reads. Nat. Methods.

[35] Simpson JT (2017). Detecting DNA cytosine methylation using nanopore sequencing. Nat. Methods.

[36] Lim KB (2007). Characterization of the centromere and peri-centromere retrotransposons in *Brassica rapa* and their distribution in related *Brassica* species. Plant J..

[37] Wang G-X (2019). ChIP-cloning analysis uncovers centromere-specific retrotransposons in *Brassica nigra* and reveals their rapid diversification in *Brassica* allotetraploids. Chromosoma.

[38] Kronmiller BA, Wise RP (2008). TEnest: automated chronological annotation and visualization of nested plant transposable elements. Plant Physiol..

[39] Lysak MA, Mandáková T, Schranz MEJCoipb (2016). Comparative paleogenomics of crucifers: ancestral genomic blocks revisited. Curr. Opin. Plant Biol..

[40] Cheng F (2012). Biased gene fractionation and dominant gene expression among the subgenomes of *Brassica rapa*. PLoS ONE.

[41] Eichler EE, Sankoff DJ (2003). Structural dynamics of eukaryotic chromosome evolution. Science.

[42] Yancopoulos S, Attie O, Friedberg RJB (2005). Efficient sorting of genomic permutations by translocation, inversion and block interchange. Bioinformatics.

[43] Michael TP (2018). High contiguity *Arabidopsis thaliana* genome assembly with a single nanopore flow cell. Nat. Commun..

[44] Gabur I, Chawla HS, Snowdon RJ, Parkin IAP (2018). Connecting genome structural variation with complex traits in crop plants. Theor. Appl. Genet..

[45] Cameron DL, Di Stefano L, Papenfuss AT (2019). Comprehensive evaluation and characterisation of short read general-purpose structural variant calling software. Nat. Commun..

[46] De Coster, W. et al. Structural variants identified by Oxford Nanopore PromethION sequencing of the human genome. *Genome Res.* **29**, 1178–1187 (2019). 10.1101/gr.244939.118 PMC6633254 31186302

[47] van Dijk EL, Jaszczyszyn Y, Naquin D, Thermes C (2018). The third revolution in sequencing technology. Trends Genet..

[48] Zhang W, Lee H-R, Koo D-H, Jiang J (2008). Epigenetic modification of centromeric chromatin: hypomethylation of DNA sequences in the CENH3-associated chromatin in *Arabidopsis thaliana* and maize. Plant Cell.

[49] Kronmiller BA, Wise RPJ (2009). Computational finishing of large sequence contigs reveals interspersed nested repeats and gene islands in the rf1-associated region of maize. Plant Physiol..

[50] Gao, D., Jiang, N., Wing, R. A., Jiang, J. & Jackson, S. A. Transposons play an important role in the evolution and diversification of centromeres among closely related species. *Front. Plant Sci.* **6**, 216 (2015). 10.3389/fpls.2015.00216 PMC4387472 25904926

[51] Zhang HB, Zhao X, Ding X, Paterson AH, Wing RA (1995). Preparation of megabase‐size DNA from plant nuclei. Plant J..

[52] Allen G, Flores-Vergara M, Krasynanski S, Kumar S, Thompson WJNp (2006). A modified protocol for rapid DNA isolation from plant tissues using cetyltrimethylammonium bromide. Nat. Protoc..

[53] De Coster W, D’Hert S, Schultz DT, Cruts M, Van Broeckhoven C (2018). NanoPack: visualizing and processing long-read sequencing data. Bioinformatics.

[54] Li B, Dewey CNJ (2011). RSEM: accurate transcript quantification from RNA-Seq data with or without a reference genome. BMC Bioinformatics.

[55] Sun H, Ding J, Piednoël M, Schneeberger K (2018). findGSE: estimating genome size variation within human and *Arabidopsis* using *k*-mer frequencies.. Bioinformatics.

[56] Li H (2016). Minimap and miniasm: fast mapping and de novo assembly for noisy long sequences. Bioinformatics.

[57] Moll KM (2017). Strategies for optimizing BioNano and Dovetail explored through a second reference quality assembly for the legume model, *Medicago truncatula*. BMC Genomics.

[58] He Z, Bancroft IJ (2018). Organization of the genome sequence of the polyploid crop species *Brassica juncea*. Nat. Genet..

[59] Ramchiary N (2007). Mapping of yield influencing QTL in *Brassica juncea*: implications for breeding of a major oilseed crop of dryland areas. Theor. Appl. Genet..

[60] Guo S (2012). A genetic linkage map of *Brassica carinata* constructed with a doubled haploid population. Theor. Appl. Genet..

[61] Lagercrantz U, Lydiate DJJ (1995). RFLP mapping in *Brassica nigra* indicates differing recombination rates in male and female meioses. Genome.

[62] Stanke M, Waack S (2003). Gene prediction with a hidden Markov model and a new intron submodel. Bioinformatics.

[63] Camacho C (2009). BLAST+: architecture and applications. BMC Bioinformatics.

[64] Langmead B, Salzberg SL (2012). Fast gapped-read alignment with Bowtie 2. Nat. Methods.

[65] Campbell MS, Holt C, Moore B, Yandell M (2014). Genome annotation and curation using MAKER and MAKER‐P. Curr. Protoc. Bioinformatics.

[66] Haas BJ (2003). Improving the *Arabidopsis* genome annotation using maximal transcript alignment assemblies. Nucleic Acids Res..

[67] Tarailo‐Graovac M, Chen NJ (2009). Using RepeatMasker to identify repetitive elements in genomic sequences. Curr. Protoc. Bioinformatics.

[68] Schelfhout CJ, Snowdon R, Cowling WA, Wroth JM (2004). A PCR based B-genome-specific marker in *Brassica* species. Theor. Appl. Genet..

[69] Ellinghaus D, Kurtz S, Willhoeft U (2008). LTRharvest, an efficient and flexible software for de novo detection of LTR retrotransposons. BMC Bioinformatics.

[70] Xu Z, Wang H (2007). LTR_FINDER: an efficient tool for the prediction of full-length LTR retrotransposons. Nucleic Acids Res..

[71] Ou S, Jiang N (2018). LTR_retriever: a highly accurate and sensitive program for identification of long terminal repeat retrotransposons. Plant Physiol..

[72] Neumann P, Novák P, Hoštáková N, Macas J (2019). Systematic survey of plant LTR-retrotransposons elucidates phylogenetic relationships of their polyprotein domains and provides a reference for element classification. Mobile DNA.

[73] Haas BJ, Delcher AL, Wortman JR, Salzberg SL (2004). DAGchainer: a tool for mining segmental genome duplications and synteny. Bioinformatics.

[74] Qiao X (2019). Gene duplication and evolution in recurring polyploidization–diploidization cycles in plants. Genome Biol..

[75] Larkin MA (2007). Clustal W and Clustal X version 2.0. Bioinformatics.

[76] Capella-Gutierrez S, Silla-Martinez JM, Gabaldon T (2009). trimAl: a tool for automated alignment trimming in large-scale phylogenetic analyses. Bioinformatics.

[77] Smith SA, Dunn CW (2008). Phyutility: a phyloinformatics tool for trees, alignments and molecular data. Bioinformatics.

[78] Stamatakis A (2014). RAxML version 8: a tool for phylogenetic analysis and post-analysis of large phylogenies. Bioinformatics.

[79] Letunic I, Bork P (2019). Interactive Tree Of Life (iTOL) v4: recent updates and new developments. Nucleic Acids Res..

[80] Tannier E, Zheng C, Sankoff DJ (2009). Multichromosomal median and halving problems under different genomic distances. BMC Bioinformatics.

[81] Xu, A. W. DCJ median problems on linear multichromosomal genomes: graph representation and fast exact solutions. In *RECOMB International Workshop on Comparative Genomics* (Eds Ciccarelli, F. D. & Miklós, I.) 70–83 (Springer, 2009).

[82] Suyama M, Torrents D, Bork P (2006). PAL2NAL: robust conversion of protein sequence alignments into the corresponding codon alignments. Nucleic Acids Res..

[83] Yang Z (2007). PAML 4: phylogenetic analysis by maximum likelihood. Mol. Biol. Evol..

[84] Goldman N, Yang Z (1994). A codon-based model of nucleotide substitution for protein-coding DNA sequences. Mol. Biol. Evol..

[85] Li P (2016). RGAugury: a pipeline for genome-wide prediction of resistance gene analogs (RGAs) in plants. BMC Genomics.

[86] Zheng Y (2016). iTAK: a program for genome-wide prediction and classification of plant transcription factors, transcriptional regulators, and protein kinases. Mol. Plant.

[87] Xi Y, Li W (2009). BSMAP: whole genome bisulfite sequence MAPping program. BMC Bioinformatics.

[88] Rice P, Longden I, Bleasby A (2000). EMBOSS: the European Molecular Biology Open Software Suite. Trends Genet..

[89] Krzywinski M (2009). Circos: an information aesthetic for comparative genomics. Genome Res..

