# Supplementary material for: A high-contiguity Brassica nigra genome localizes active centromeres and defines the ancestral Brassica genome
Source: Nat Plants. 2020 Aug 10;6(8):929–41. doi: 10.1038/s41477-020-0735-y (PMC7419231; doi:10.1038/s41477-020-0735-y)
Supplement: Supplementary file 1 — Supplementary Figs. 1–25. [file 41477_2020_735_MOESM1_ESM.pdf]

nature research https://doi.org/10.1038/s41477-020-0735-y

Supplementary information

A high-contiguity *Brassica nigra* genome localizes active centromeres and defines the ancestral *Brassica* genome

In the format provided by the authors and unedited

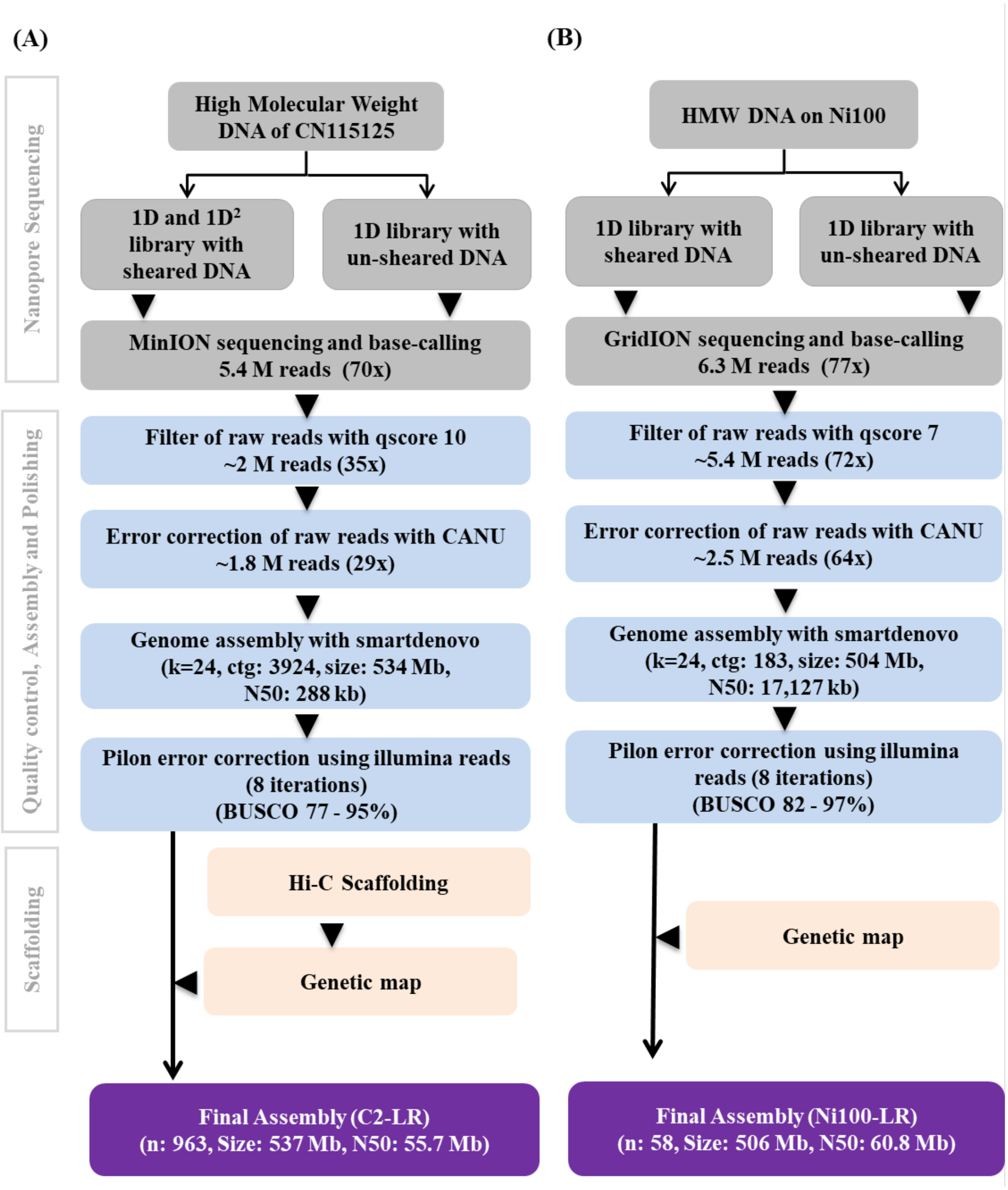

Supplementary Figure 1. ONT assembly schema for *B. nigra* CN115125 (A) and Ni100 (B) genomes

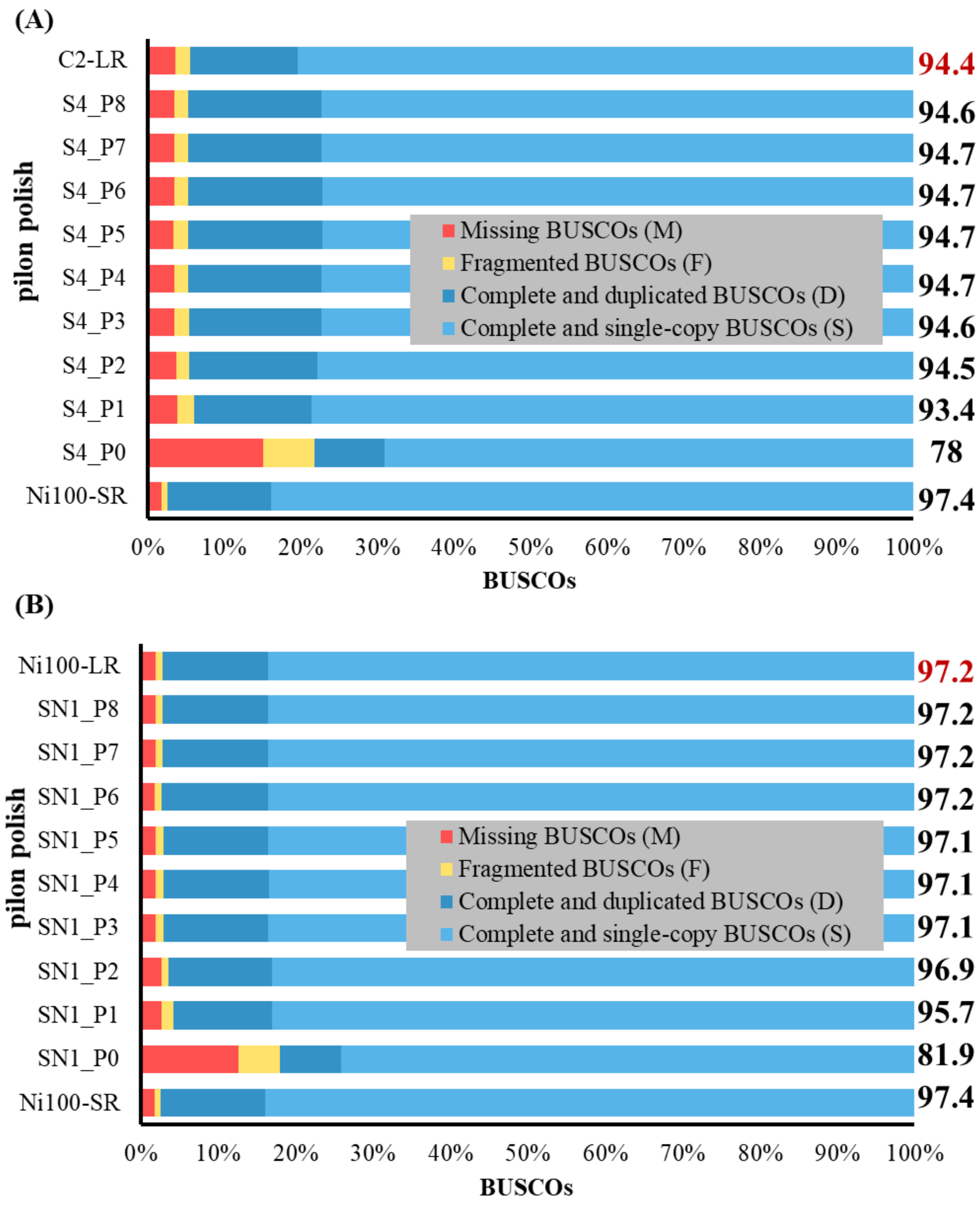

Supplementary Figure 2. Quality assessment of the *B. nigra* assemblies *B. nigra* CN115125 (A) and Ni100 (B) by BUSCO analysis. Assemblies before Pilon polishing (P0) and after 8 rounds of Pilon (P1-P8). Ni100-Short-read assembly (Ni100-SR) was used as reference and C2-LR and Ni100-LR are the final-assemblies of the respective genomes.

(A)

Assembly stats	Input Assembly	Scaffolding	
		Chicago™	Chicago™ + Hi-C
Total Length (Mb)	536.23	536.49	536.62
L50 (scaffolds)	424	186	5
N50 (Mb)	0.29	0.605	44.582
L90 (scaffolds)	2,192	1,020	27
N90 (Mb)	0.052	0.109	0.302
Longest Scaffold (bp)	4,269,980	9,115,865	68,759,150
Number of scaffolds	3,924	2,166	967
Contig N50 (Kb)	289.82	209.63	208.98
Number of gaps	0	2,602	3,822
Percent of genome in gaps	0	0.05%	0.07%
BUSCO (%)	95	97	97

(B)

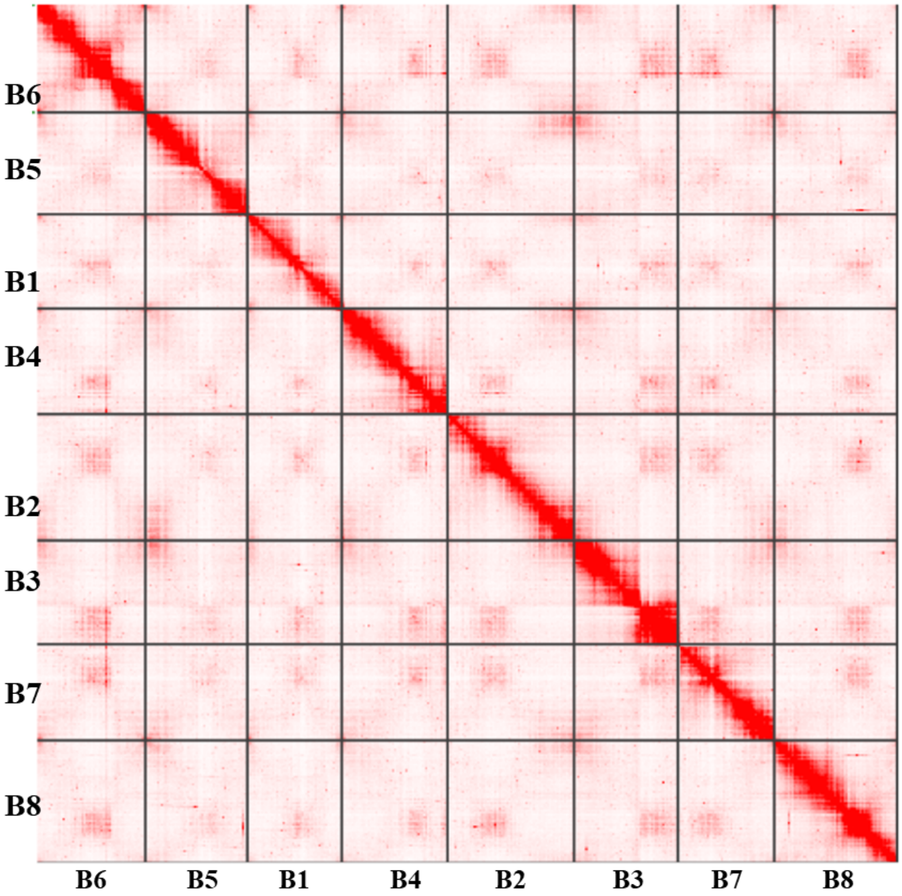

Supplementary Figure 3. Summary of Hi-C scaffolding assembly of C2-LR. (A) Summary statistics of scaffolding with Chicago™ and Hi-C data. (B) Hi-C map showing the interaction frequency of the mapping of read-pairs to the final scaffold assembly with over 1 Mb size. The color key of the heatmap indicated the frequency of the reads linked to the scaffold of each square gives the number of read pairs within that bin.

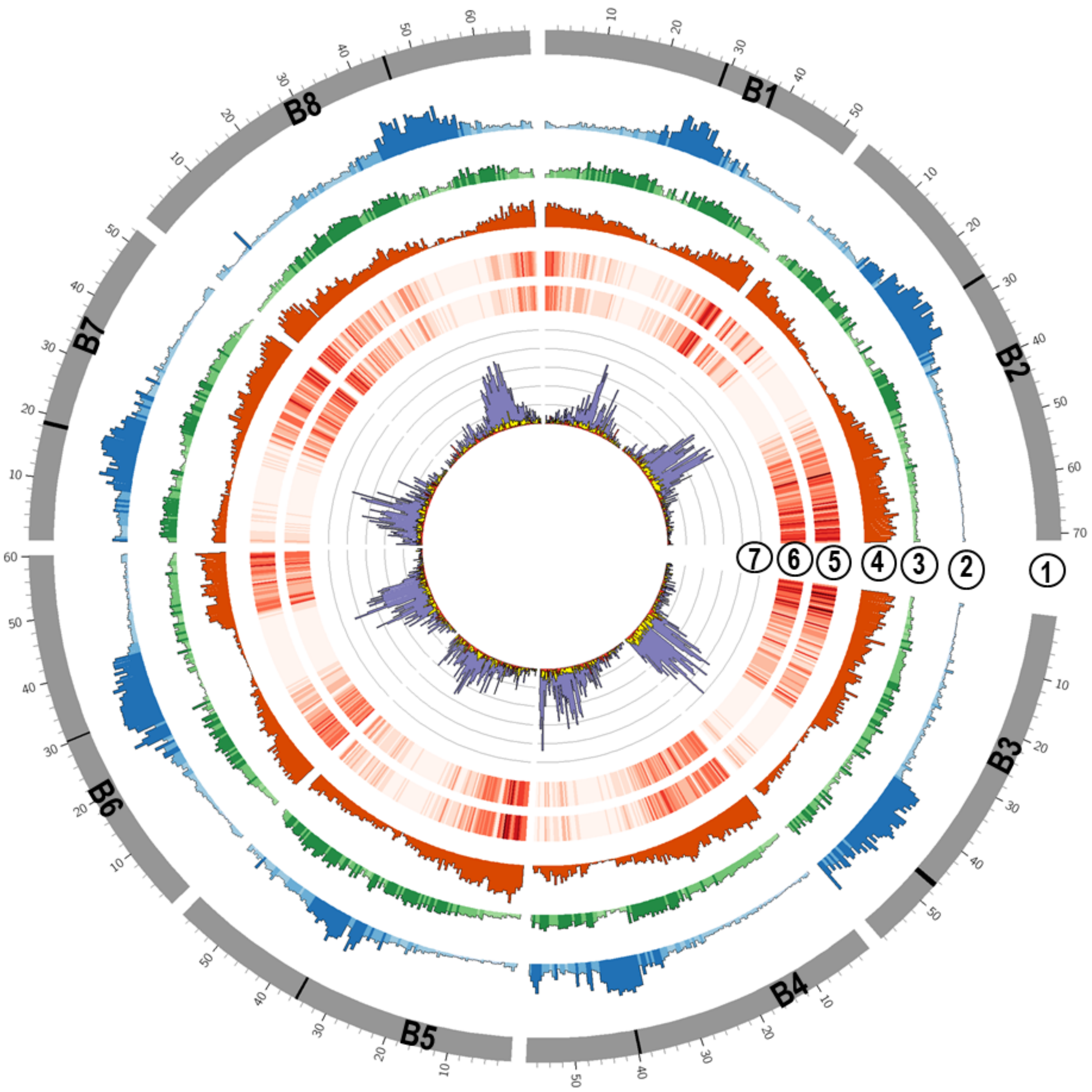

Supplementary Figure 4. Genomic features of the *B. nigra* C2-LR assembly. Tracks (1) Chromosomes with centromere, (2) Class 1 repeats (3) Class 2 repeats, (4) Gene density, (5) gene expression in TPM in leaf tissue (6) gene expression in TPM in bud tissue (7) WGBS methylation profile in leaf tissue – CG (purple), CHG (yellow), CHH (red)

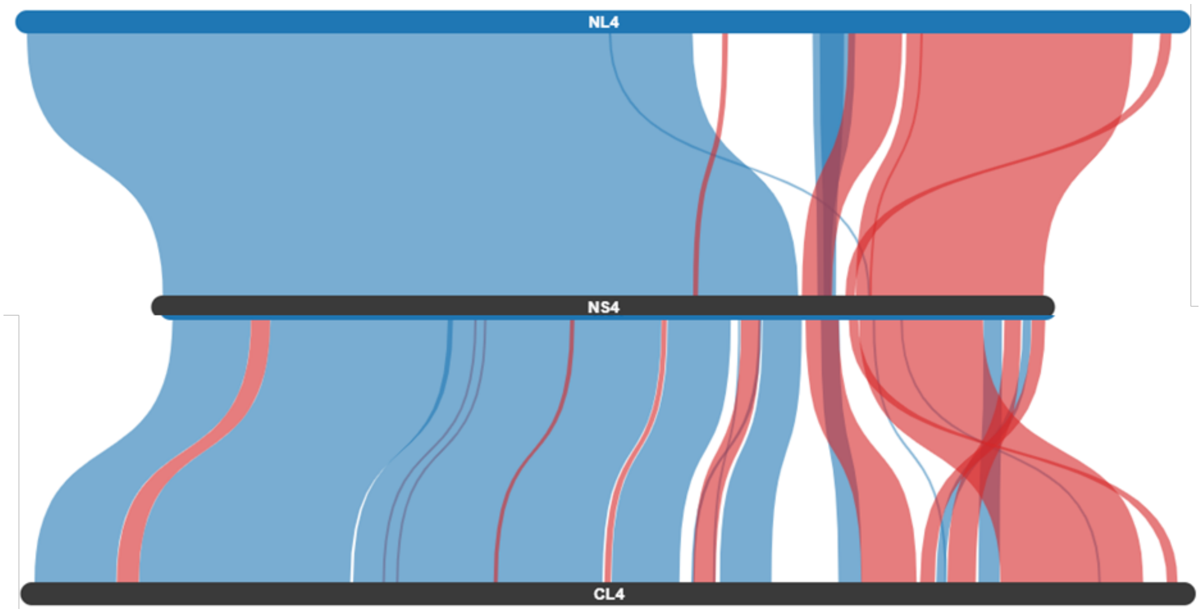

Supplementary Figure 5. Alignment of chromosome 4 from three assemblies (NL; Ni100-LR, NS; Ni100-SR and CL; C2-LR) showing large-scale structural rearrangements between the three *B. nigra* genomes. Syntenic regions shaded in red represent inverted regions.

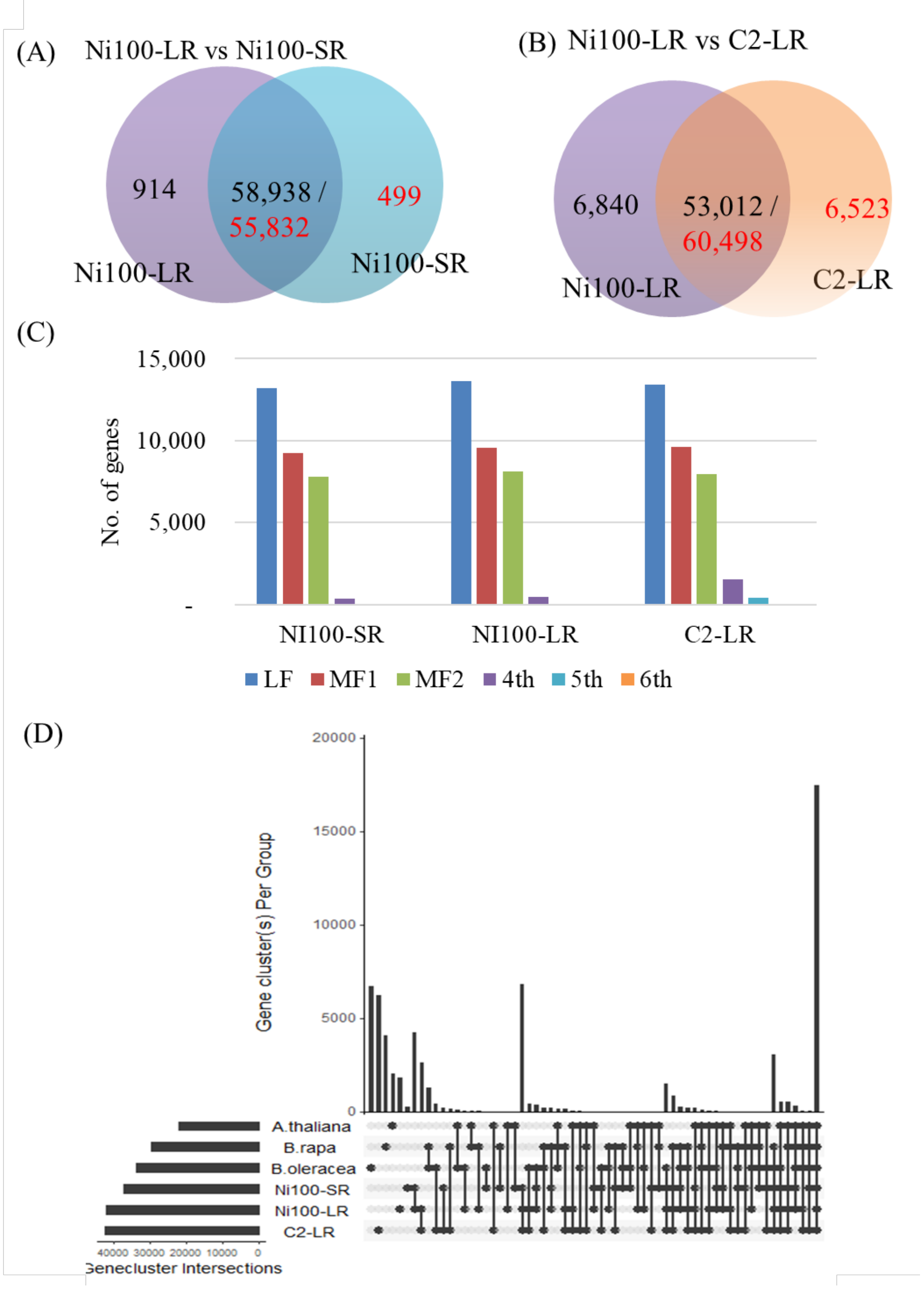

Supplementary Figure 6. Gene copy number and gene synteny between three *B. nigra* assemblies. GMAP comparison of common and unique genes between the Ni100-LR and Ni100-SR (A), and Ni100-LR and C2-LR (B) assemblies. (C) Comparison of number of syntenic genes between the three *B. nigra* genomes. (D) upsetR plot showing intersect of numbers of gene families between the three *B. nigra* genomes, *Arabidopsis thaliana*, *B. rapa* and *B. oleracea*.

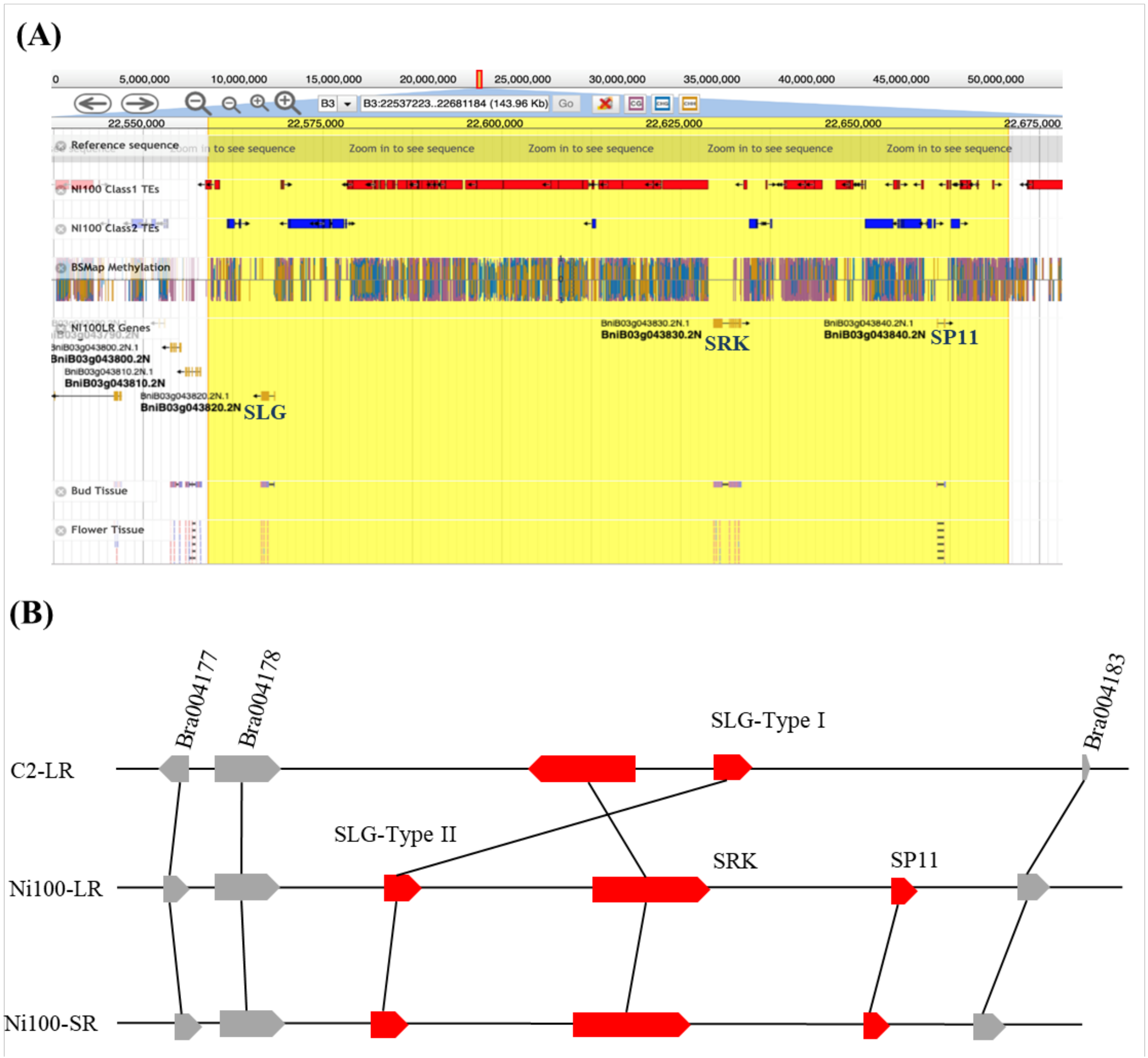

Supplementary Figure 7. S-locus of *B. nigra* genomes.(A) A Jbrowse view showing the highly repeat-rich S-locus region consist of three genes (SLG, SRK and SP11) spanned over 143 kb region from *B. nigra* Ni100 LR assembly. (B) Comparison of S-locus between three *B. nigra* assemblies showing C2-LR having a different rearrangement and missing the SP11 gene.

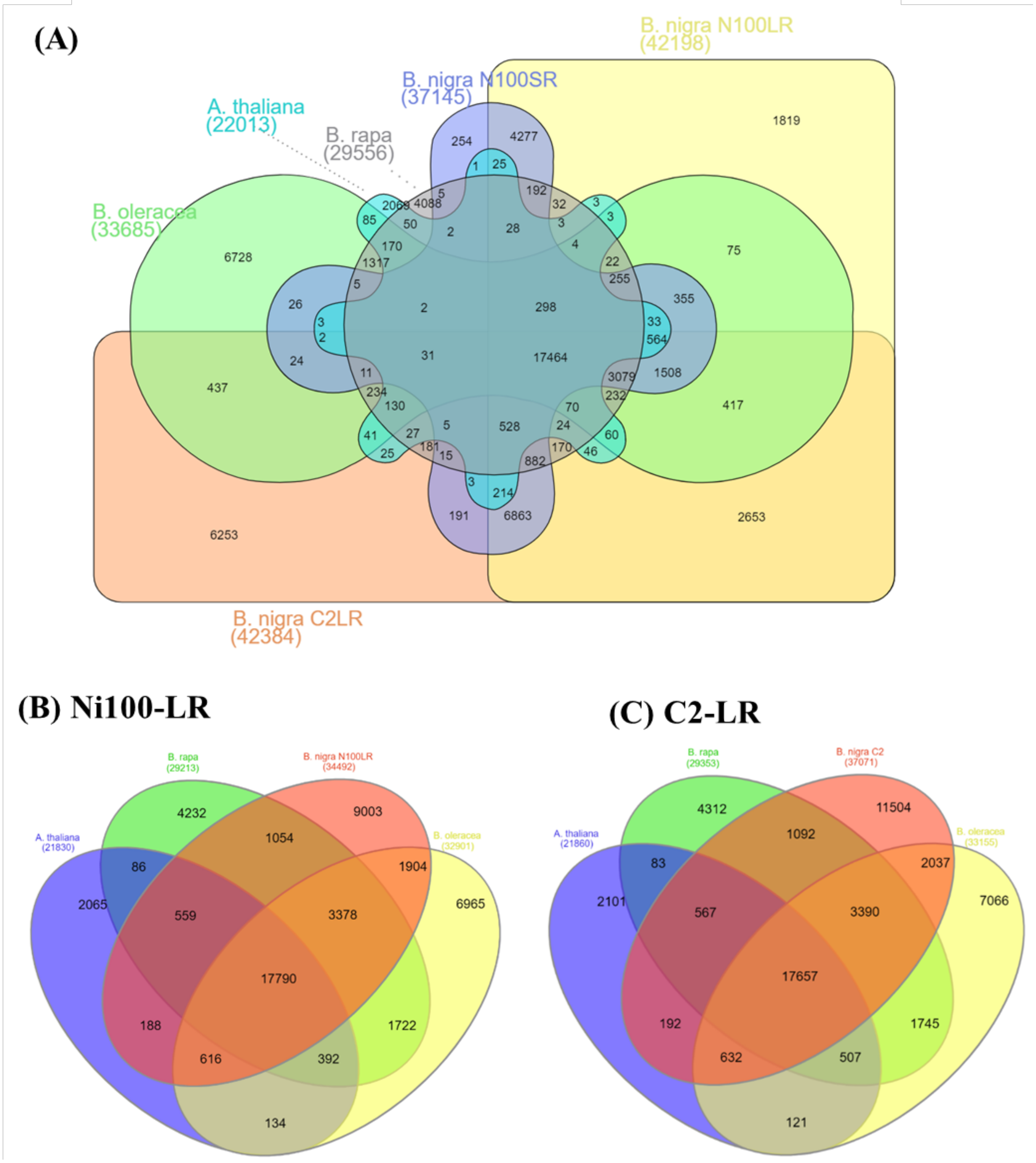

Supplementary Figure 8. Venn diagram showing orthologous gene families based on (A) Three *B. nigra, Arabidopsis thaliana*, *B. rapa* and *B. oleracea genomes*. (B) *B. nigra Ni100-LR,* against *A. thaliana*, *B. rapa,* and *B. oleracea*. (C) *B. nigra C2-LR,* against *A. thaliana*, *B. rapa*, and *B. oleracea.* Numbers correspond to number of gene clusters.

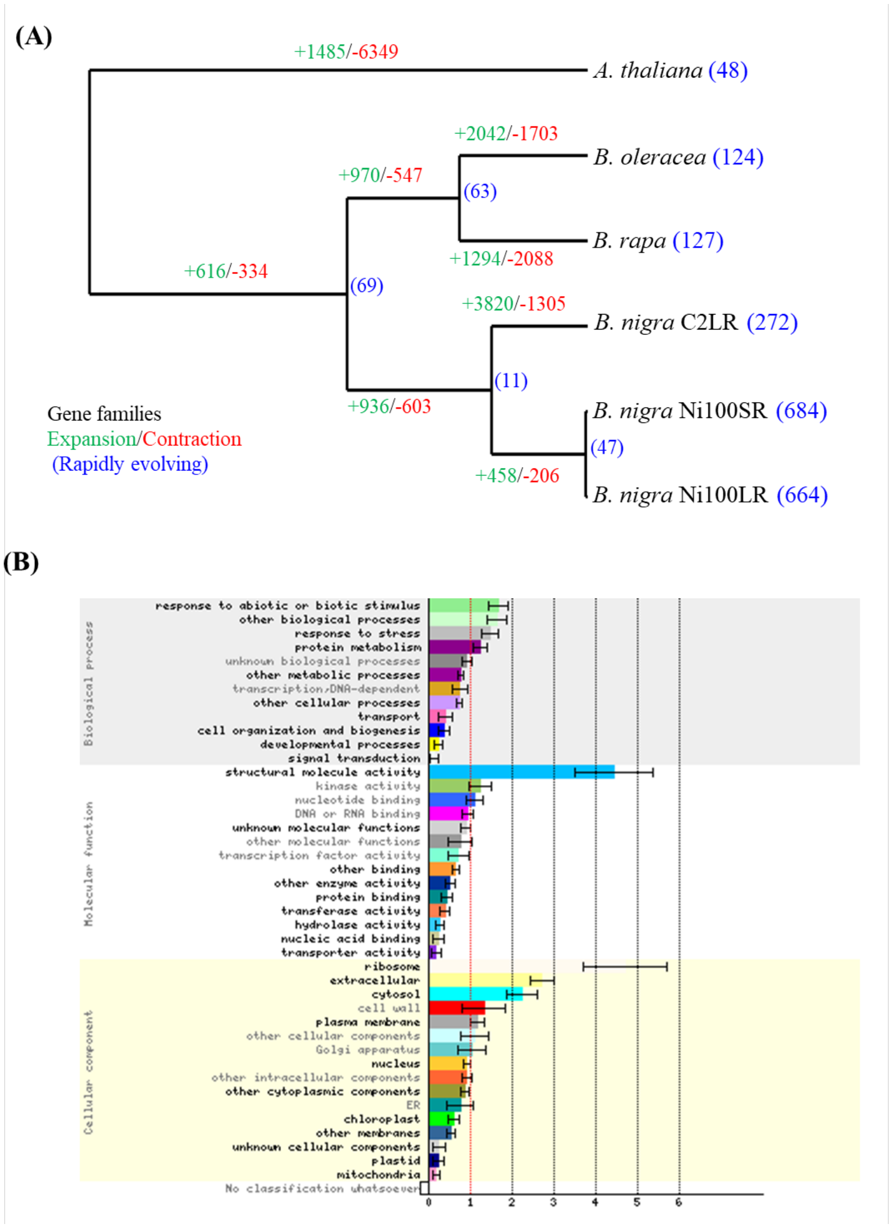

Supplementary Figure 9. Evolution of gene families. (A) Expansion, contraction and rapidly evolving gene families among the 6 genomes. (B) Gene ontology (GO) functional classification of rapidly evolving genes from 69 orthogroups diverging between the *B. nigra* and *B. rapa*-*B. oleracea* lineage. GO classification was completed using 242 orthologous gene IDs from Arabidopsis at the following website: http://bar.utoronto.ca/ntools/cgi-bin/ntools_classification_superviewer.cgi#class_33, which performs normalization of the classification and bootstraps the input dataset to provide a confidence estimate for the accuracy of the output. A class score for normalization is calculated and the input set is bootstrapped 100 times by sampling the input set (with repeats) and then classifying each set so generated. The standard deviation (bars) for the scores generated from the bootstrap sets is displayed along with the normalized class score.

(A)

Ni100-LR SVs - C2-LR reads against Ni100-LR assembly					
SV type/ size (kb)	<1	1 - 50	50 - 100	>100	Total
Deletion	3,184	1,855	2	18	5,059
Insertion	1,876	26	-	-	1,902
Inversion	-	17	1	-	18
Duplication	41	58	2	35	136
Translocation	66	-	-	-	66
**C2-LR SVs Ni100-LR reads against C2-LR assembly**					
Deletion	2,687	1,130	8	31	3,856
Insertion	1,879	42	-	-	1,921
Inversion	-	17	4	-	21
Duplication	40	66	5	56	167
Translocation	113	-	-	-	113

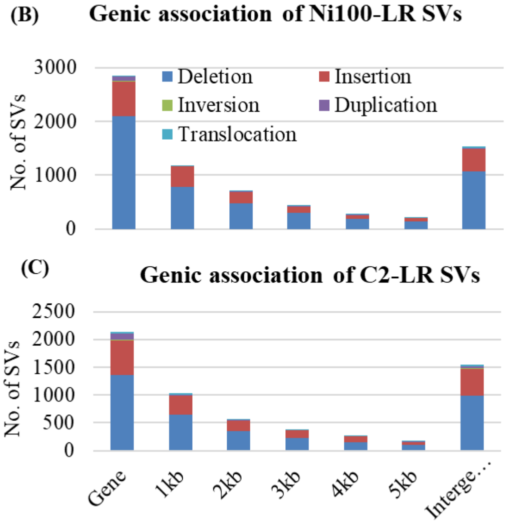

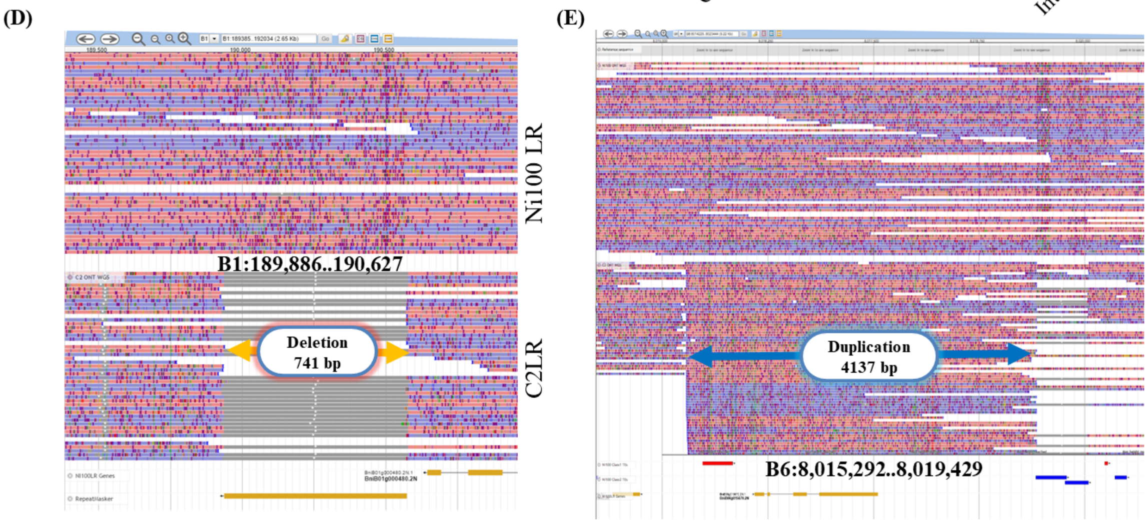

Supplementary Figure 10. Identification of Structural variants (SVs) in *B. nigra* Ni100-LR and C2-LR. (A) Summary of consensus SVs (identified by Sniffles and Picky). Association of Ni100-LR SVs (B) and C2-LR SVs (C) with gene regions and flanking DNA. Jbrowse alignments showing a deletion (741 bp) of a transposable element (D), and duplication (4137 bp) of an annotated gene in the C2-LR genome compared to Ni100 (E).

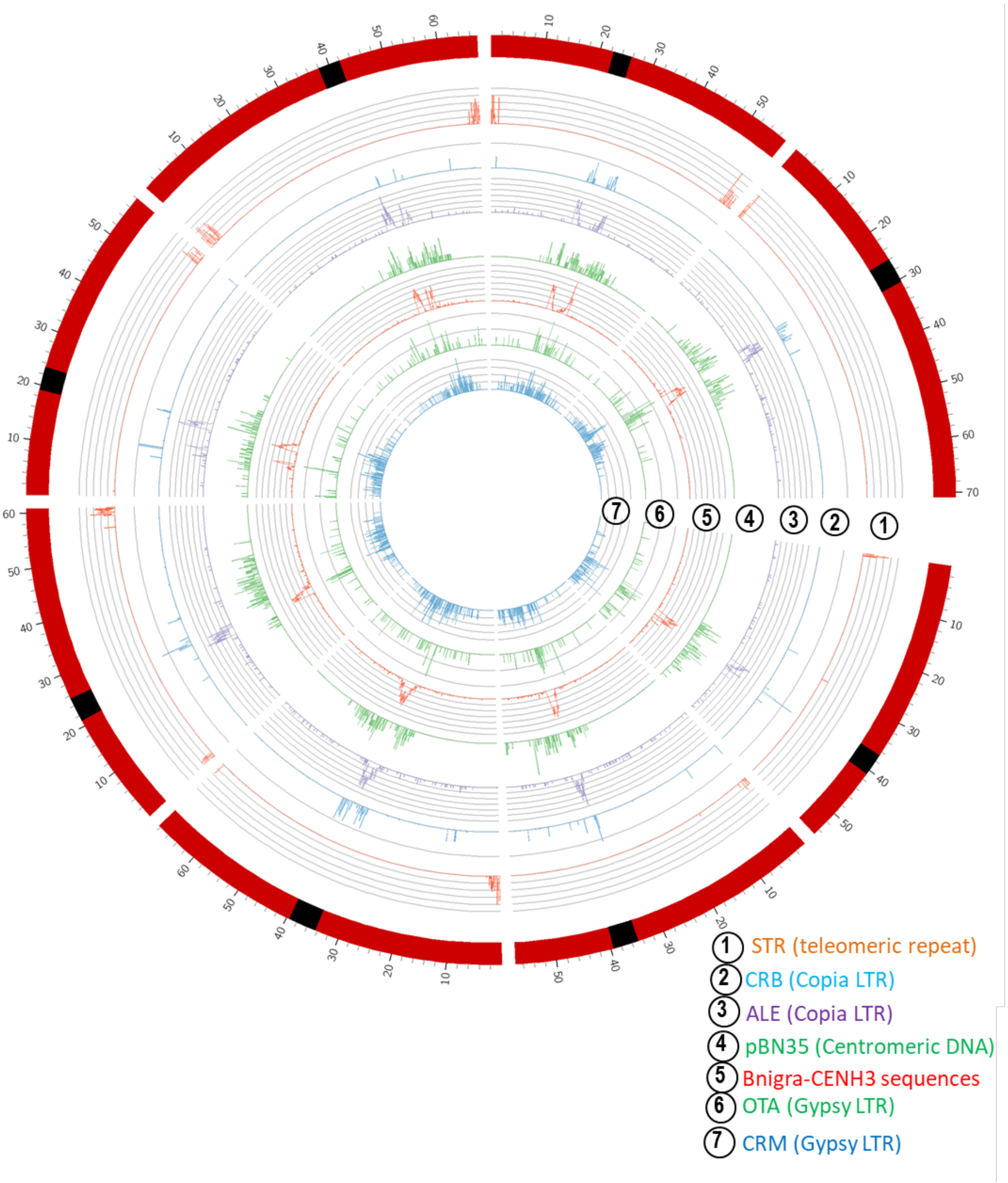

Supplementary Figure 11. Distribution of centromeric, peri-centromeric and sub-telomeric repeats in Ni100-LR.

(A)

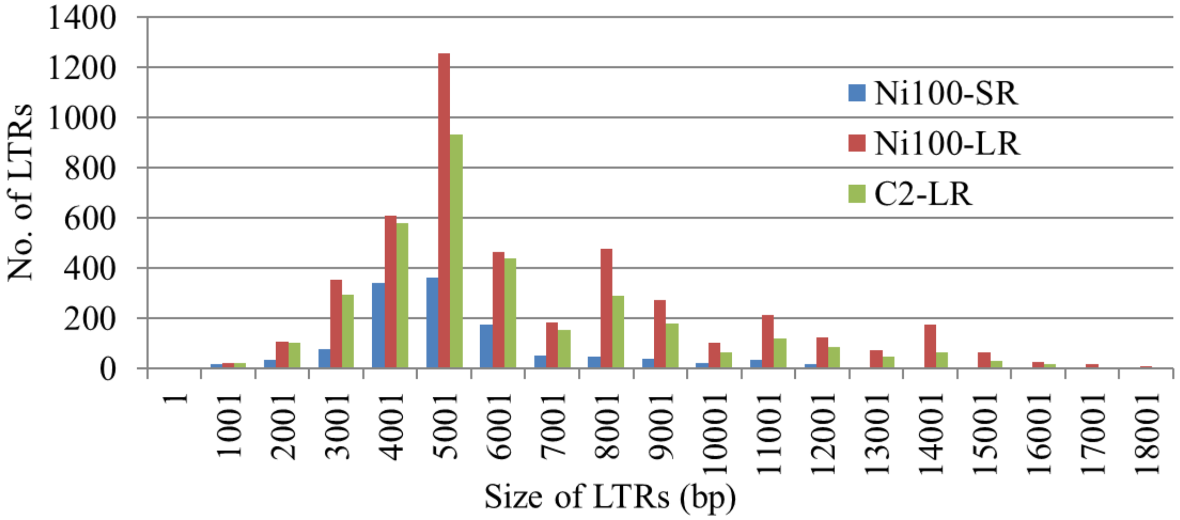

(B)

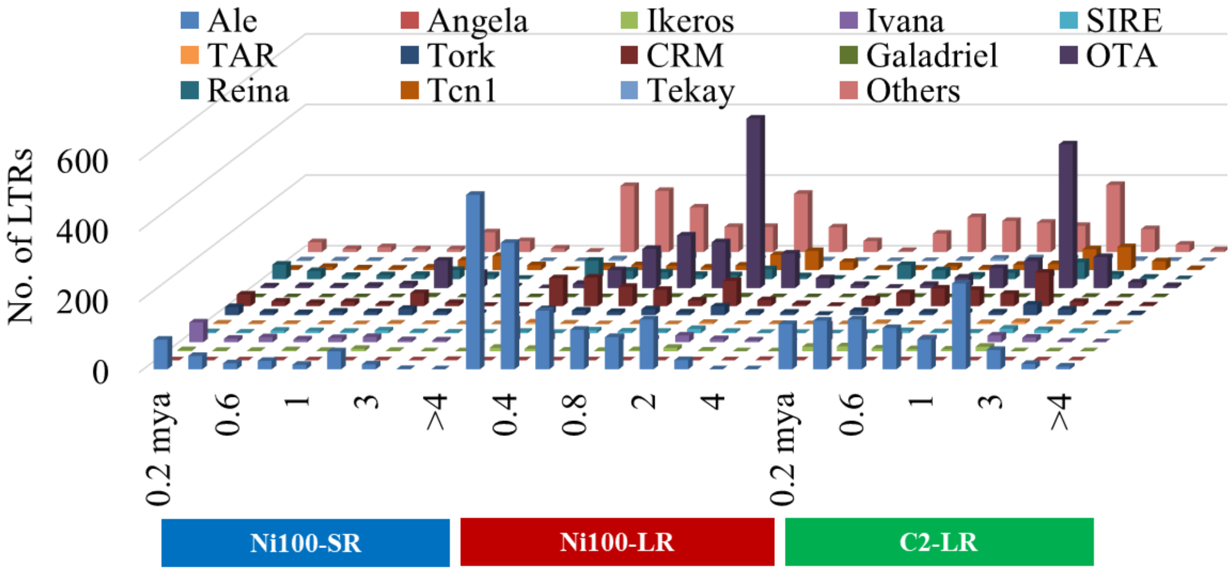

Supplementary Figure 12. Annotation of FL-LTR-RTs families in *B. nigra* genomes. (A) Size distribution of FL-LTR-RTs families. (B) Histogram showing number and age distribution of 14 FL-LTR-RTs families. Age of LTR was represented as million years ago (mya).

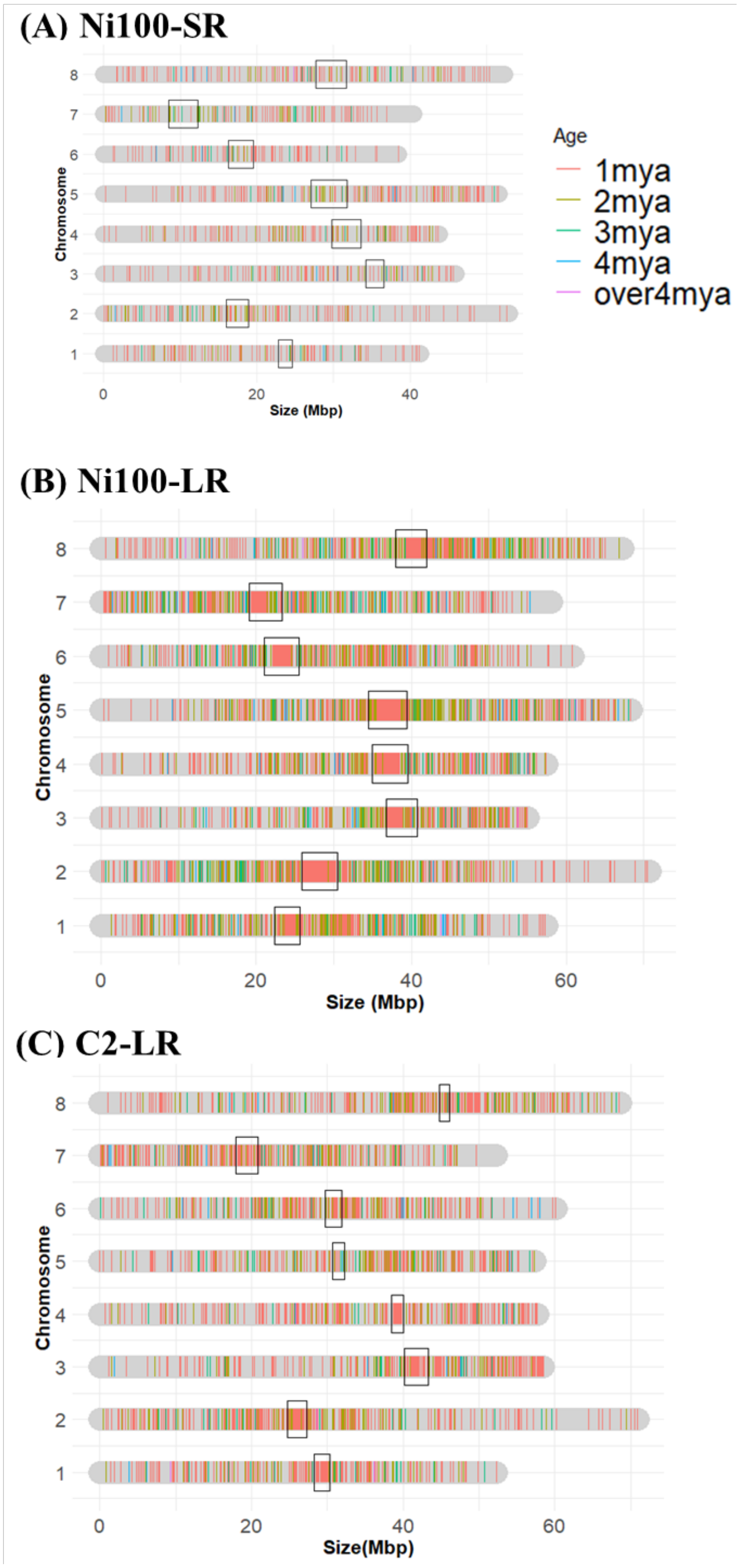

Supplementary Figure 13. Age distribution of FL-LTR-RTs in 3 *B. nigra* assemblies. Black boxes indicate the centromeric region.

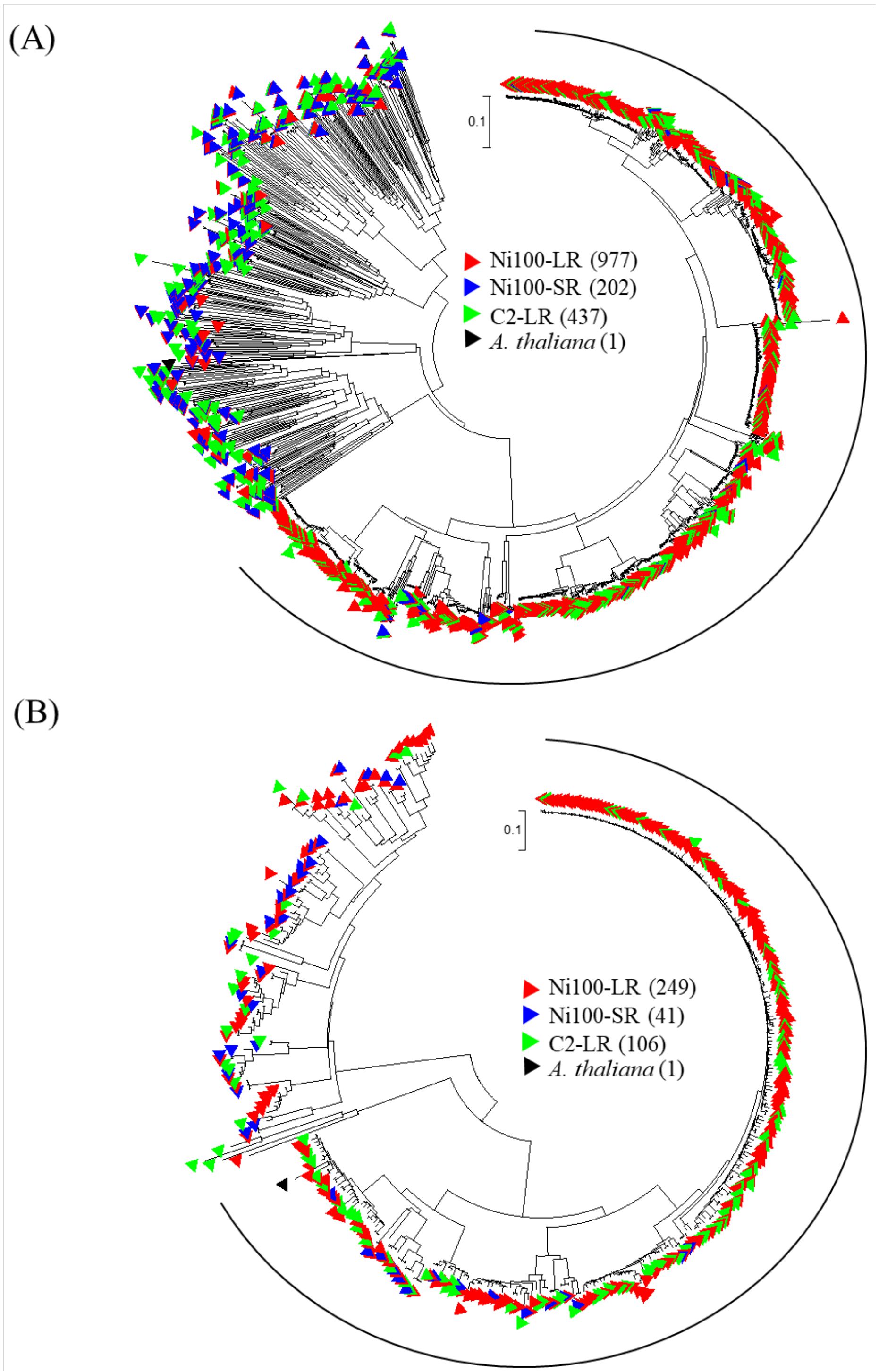

Supplementary Figure 14. Phylogenetic analysis of full-length LTRs families ALE-Copia (A) and OTA-Gypsy (B) in three *B. nigra* assemblies. Reverse-transcriptase (RT) domain sequences from three *B. nigra* genomes were aligned using clustalW followed by neighbor-joining tree construction with 500 bootstrap replications using MEGA7. Major clades are indicated by black semicircular line.

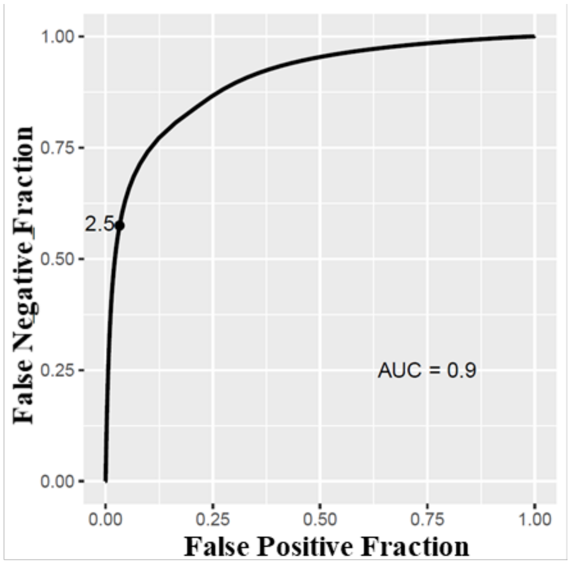

Supplementary Figure 15. Receiver operating characteristic (ROC) curve based on varying the log-likelihood ratio threshold for Ni100-LR to classify a site as methylated against filtered WGBS calls ($p <= 0.05$) based on a binomial test as described in methods.

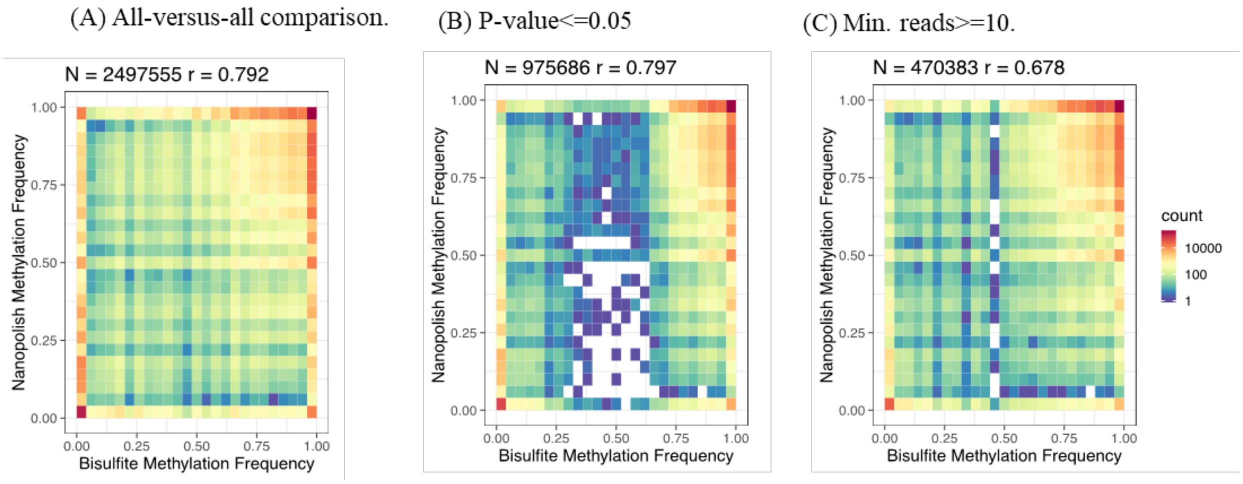

Supplementary Figure 16. Comparison of 5-methyl cytosine frequency detected by WGBS and ONT; frequency distribution plot without filtering (A) and filtering based on calls with a p-value ≤ 0.05 (based on a binomial test as described in methods) (B) or minimum ONT read depth of 10 (C).

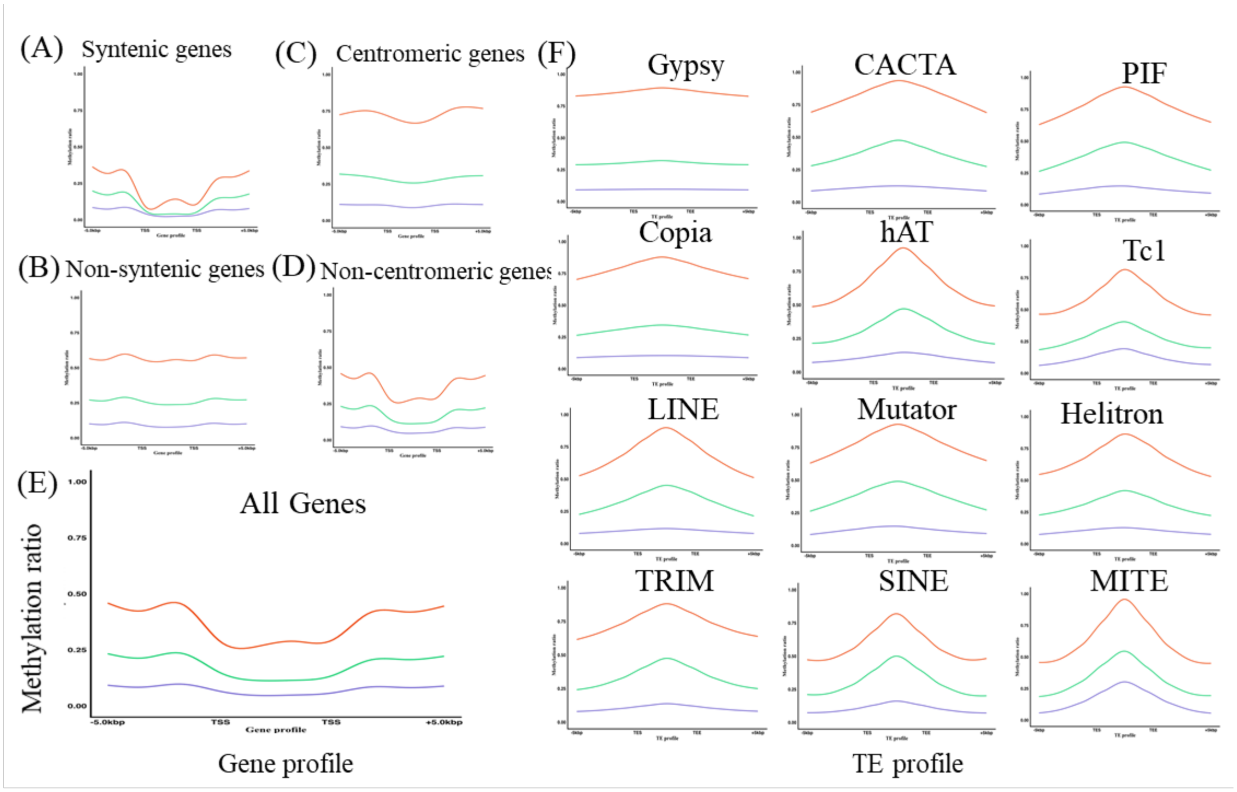

Supplementary Figure 17. Methylation landscape across the genes and transposons of *B. nigra* Ni100-LR genome from WGBS data. (A-E) Methylation levels of genes distributed across the five different regions of the genome. (F) Methylation of different classes of TEs and their 1 kb surrounding regions. DNA methylation profiles of gene and repeat features is shown in context of CpG (red), CHG (green) and CHH (blue).

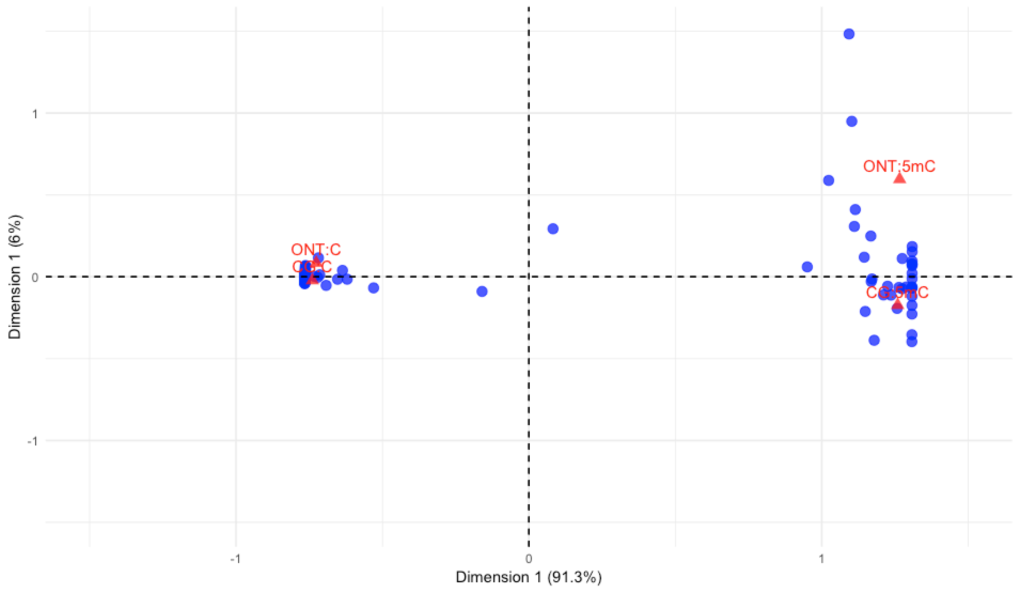

Supplementary Figure 18. Comparison of differentially methylated CG islands identified using WGBS calls (CG: C or CG:5mC) and Nanopolish calls (ONT:C or ONT:5mC) in the Ni100-LR genome. Extent of agreement in DNA methylation status at cytosine bases in CG context at CpG Islands located in the 5' regulatory regions of annotated *B. nigra* genes. The variation among these data are summarized with individual CpG Islands represented in blue and the variation among methylation status in red. DNA methylation status detected by ONT and WGBS is largely in agreement as indicated by clear separation between methylated and unmethylated base calls (red) with very little distance between the methodologies. The graph highlights that there is greater correspondence when detecting unmethylated cytosines and that variation increases slightly at methylated cytosine bases, a reflection of residual error from sequence coverage in both methods. Correspondence among CpG Islands shows greater variation resulting from a variable number of cytosines analyzed in each CpG Island, where discrepancies in Island with fewer cytosine bases are amplified.

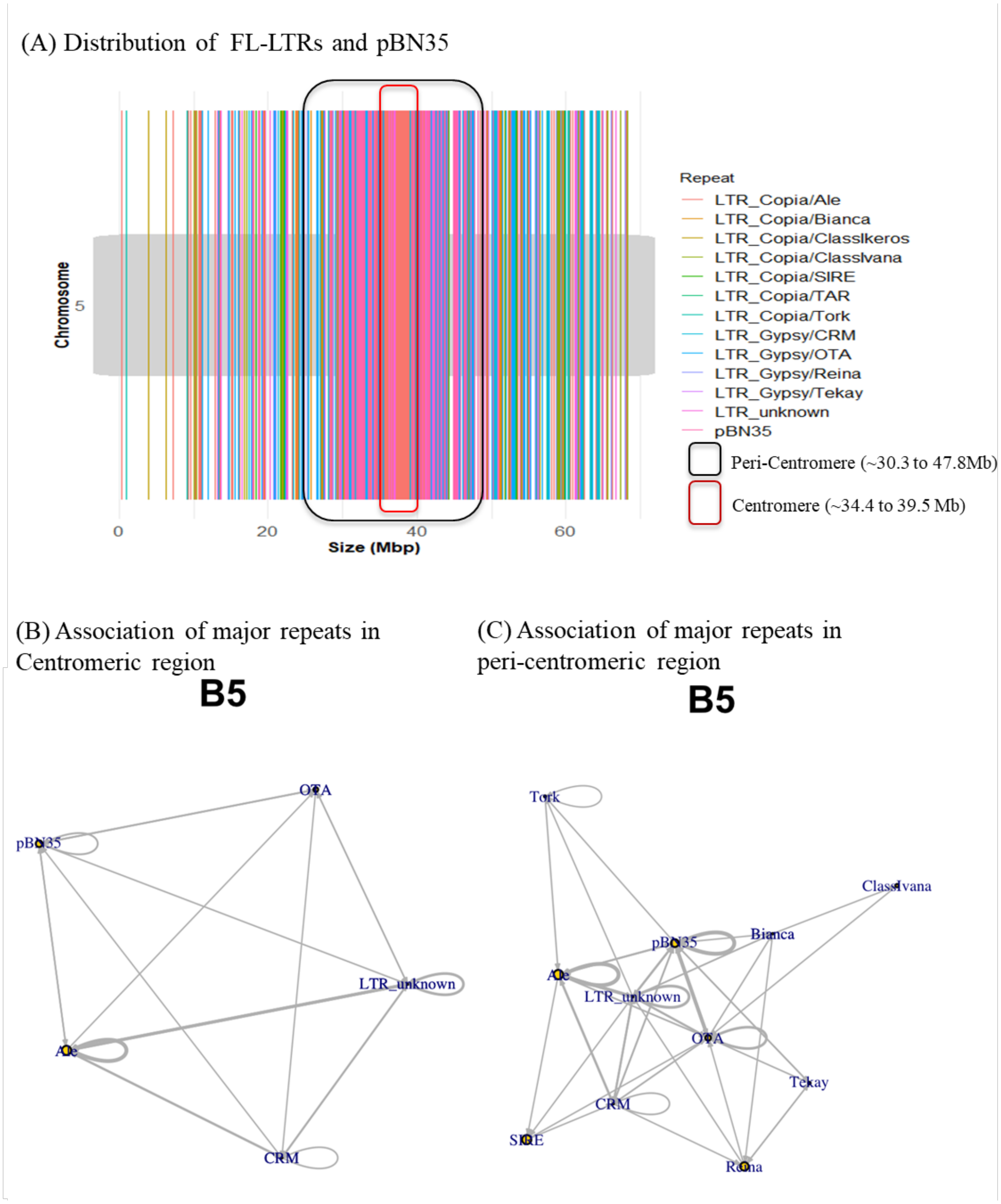

Supplementary Figure 19. Repeat distribution and complexity of the centromere of *B. nigra* Ni100-LR assembly. (A) Distribution of full length LTRs with pBN35 on the chromosome 5 centromere (red box) and peri-centromere (Black box) showing the enrichment by classes of LTRs (see Supplementary Table 20); (B, C) A graphical network representing the complexity of TEs present in the defined centromeric and pericentromeric regions of chromosome B5. The edge weighting corresponds to the frequency of neighbouring TE annotations and node size represents the occurrence of each element in the network.

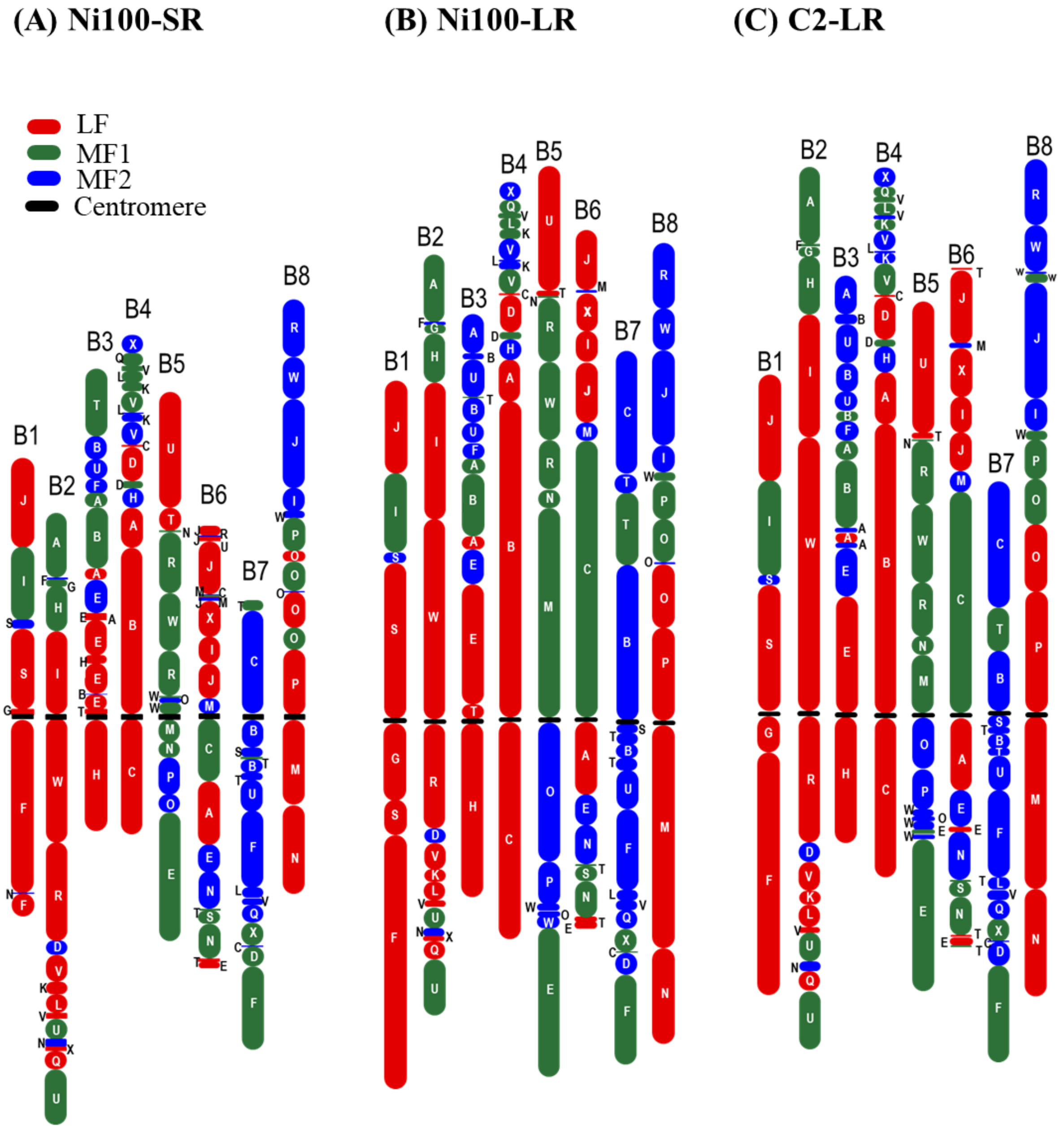

Supplementary Figure 20. Organization of triplicated genome blocks (LF, MF1, MF2) structure in the three *B. nigra* assemblies

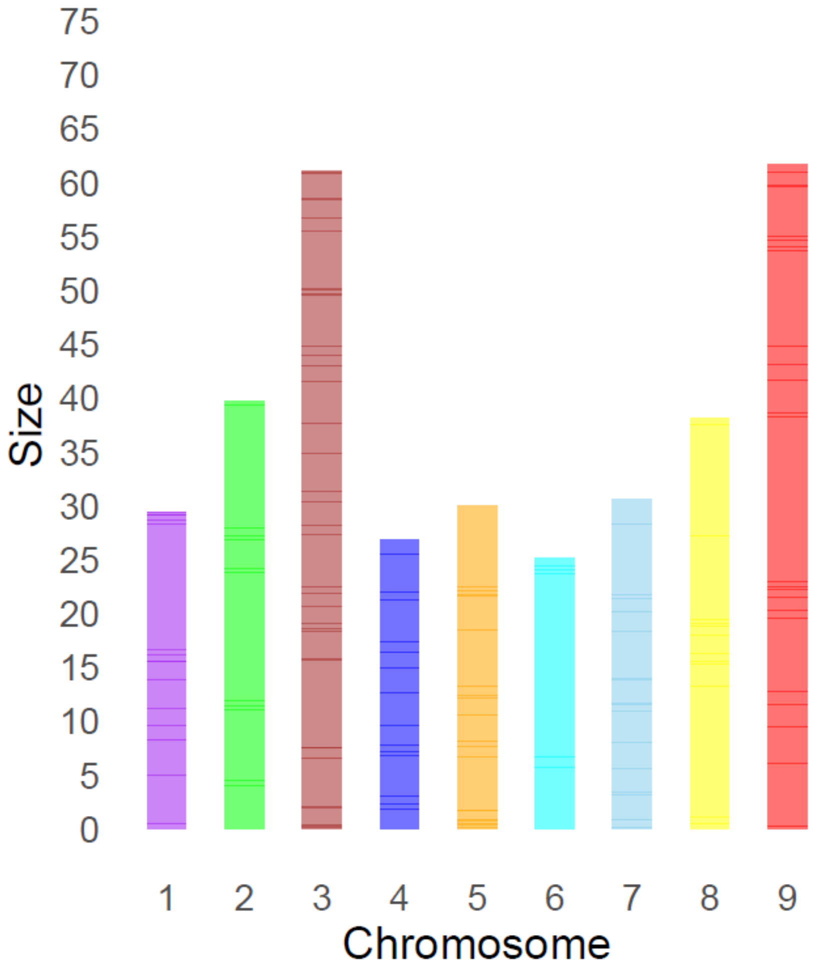

Supplementary Figure 21. Karyotype of the ancestral genome of *Brassica rapa*, *B. oleracea* and *B. nigra*

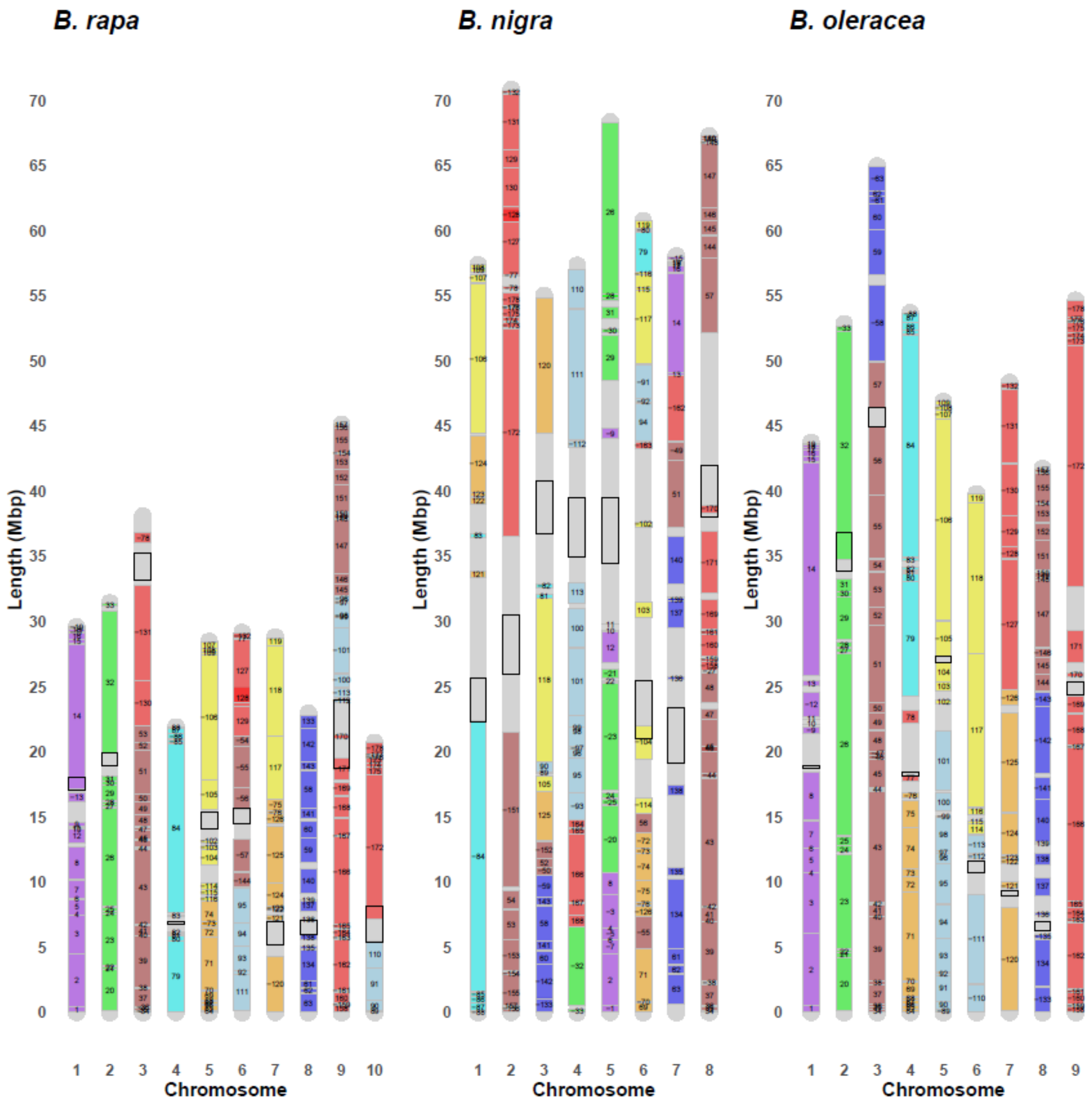

Supplementary Figure 22. Position of each ancestral block and their relative orientation on the three *Brassica* genomes. Corresponding physical position of each karyotype can be accessed from Supplementary Table 23. Black box indicates centromere position.

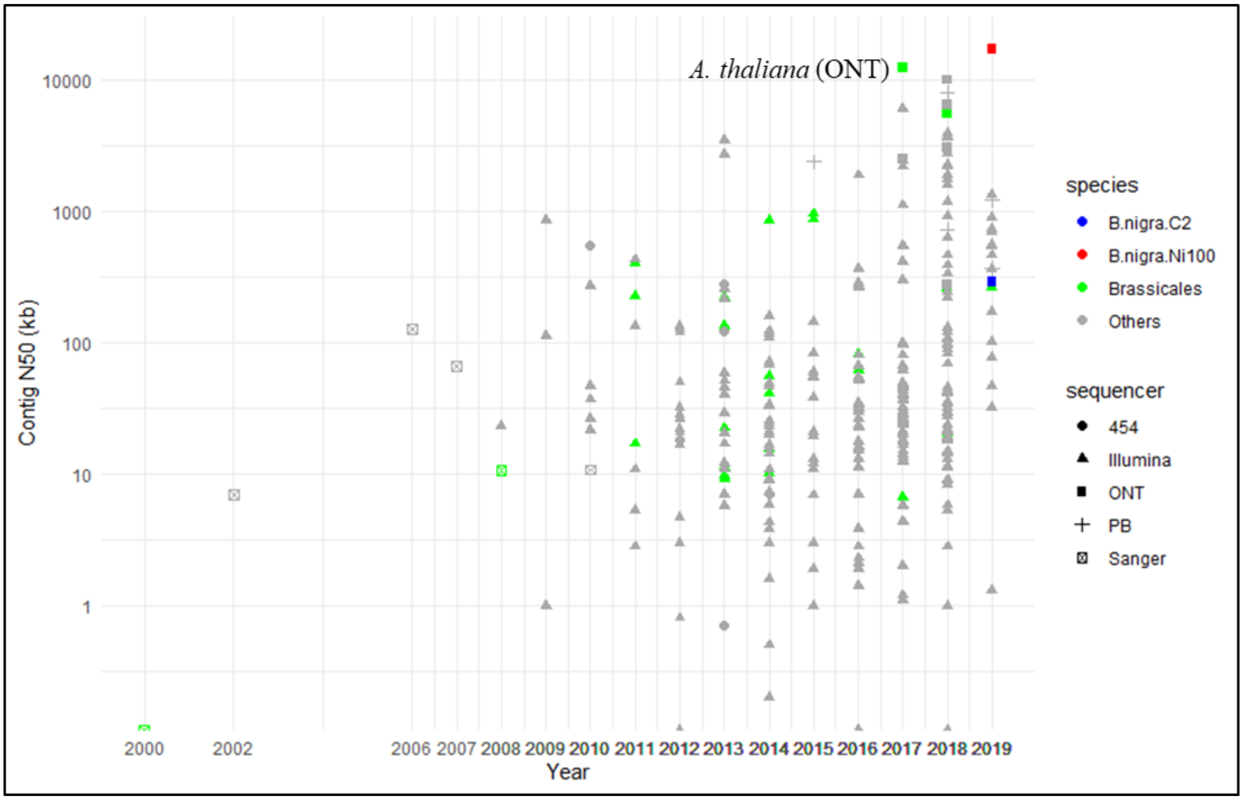

Supplementary Figure 23. Contig N50 of 324 sequenced angiosperm genomes. Genomes from the order *Brassicales* are highlighted in red. List of genomes was taken from http://www.plabipd.de/timeline_view.ep.

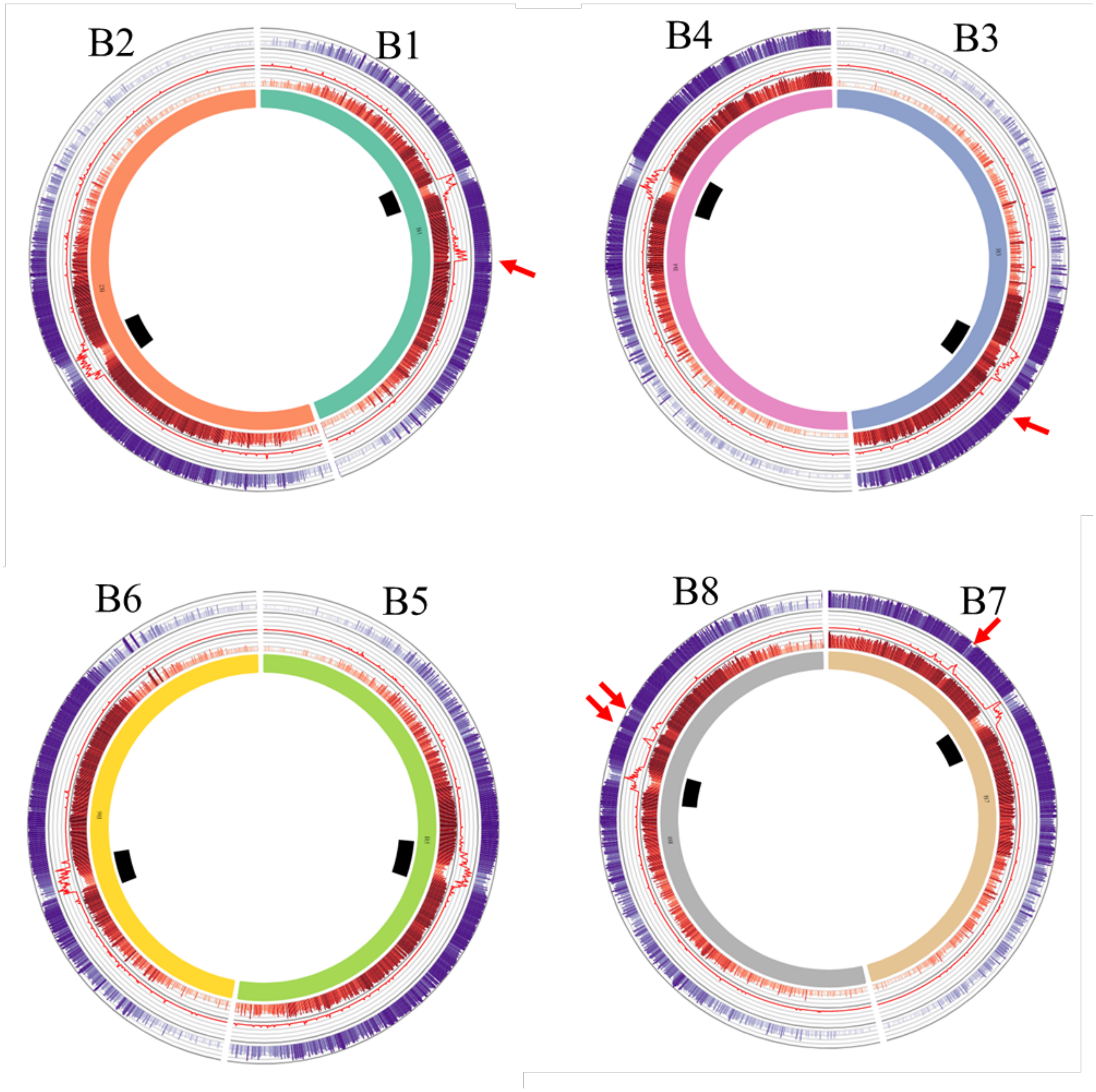

Supplementary Figure 24. Hypo-methylation in centromeres. Distribution of a centromere-specific retrotransposon with methylome in *B. nigra Ni100-LR*. Outer to inner: WGBS-CG methylome, ALE-Copia LTR and ONT-CG methylome, chromosome, centromere position (black band). Red arrow indicates additional hypo-methylated islands adjacent to centromeres. The plot was developed using AccuSyn tool (https://accusyn.usask.ca/)

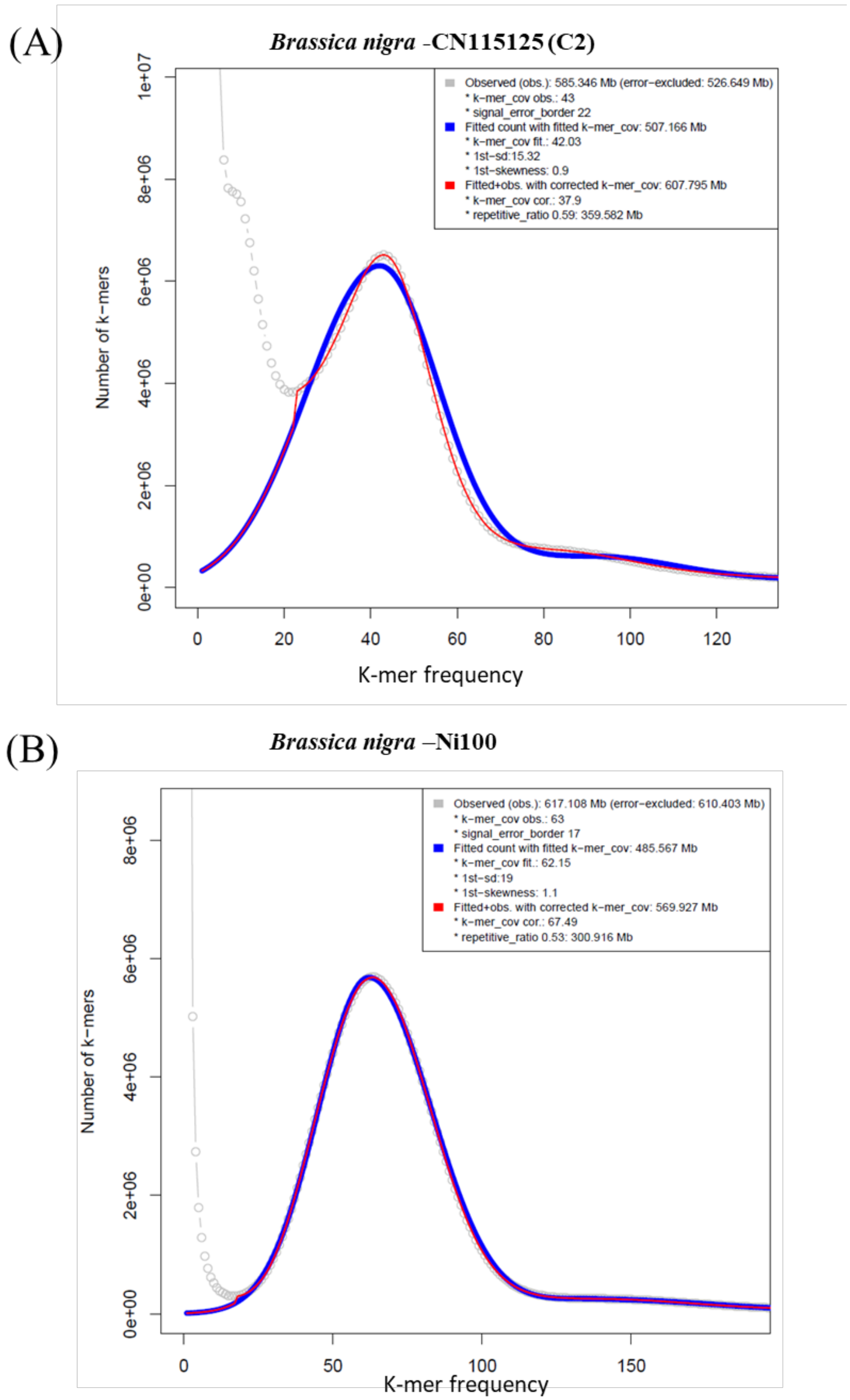

Supplementary Figure 25. K-mer based genome size estimation of *B. nigra* CN115125 and Ni100
