# Supplementary material for: A high-contiguity Brassica nigra genome localizes active centromeres and defines the ancestral Brassica genome
Source: Nat Plants. 2020 Aug 10;6(8):929–41. doi: 10.1038/s41477-020-0735-y (PMC7419231; doi:10.1038/s41477-020-0735-y)
Supplement: Supplementary file 2 — Reporting Summary [file 41477_2020_735_MOESM2_ESM.pdf]

nature research

Corresponding author(s): Isobel Parkin

Last updated by author(s): June 15th 2020

Reporting Summary

Nature Research wishes to improve the reproducibility of the work that we publish. This form provides structure for consistency and transparency in reporting. For further information on Nature Research policies, see Authors & Referees and the Editorial Policy Checklist.

Statistics

For all statistical analyses, confirm that the following items are present in the figure legend, table legend, main text, or Methods section.

n/a	Confirmed	
☐	☒	The exact sample size (n) for each experimental group/condition, given as a discrete number and unit of measurement
☒	☐	A statement on whether measurements were taken from distinct samples or whether the same sample was measured repeatedly
☐	☒	The statistical test(s) used AND whether they are one- or two-sided<br>*Only common tests should be described solely by name; describe more complex techniques in the Methods section.*
☒	☐	A description of all covariates tested
☒	☐	A description of any assumptions or corrections, such as tests of normality and adjustment for multiple comparisons
☐	☒	A full description of the statistical parameters including central tendency (e.g. means) or other basic estimates (e.g. regression coefficient) AND variation (e.g. standard deviation) or associated estimates of uncertainty (e.g. confidence intervals)
☐	☒	For null hypothesis testing, the test statistic (e.g. F, t, r) with confidence intervals, effect sizes, degrees of freedom and P value noted<br>*Give P values as exact values whenever suitable.*
☐	☒	For Bayesian analysis, information on the choice of priors and Markov chain Monte Carlo settings
☒	☐	For hierarchical and complex designs, identification of the appropriate level for tests and full reporting of outcomes
☐	☒	Estimates of effect sizes (e.g. Cohen's d, Pearson's r), indicating how they were calculated

Our web collection on statistics for biologists contains articles on many of the points above.

Software and code

Policy information about availability of computer code

Data collection	MinKnowv1.4.2 and v2.0 and albacorev1.1.2 - nanopore sequencing
Data analysis	Trimmomatic v0.32 - sequence clean up; RSEM v1.2.31 - expression analyses; Jellyfish v2.2.6 - kmer frequency; CANU v1.6 - error correction; SmartDenovo v1.0 - genome assembly; Pilon1.22 - error correction; BUSCO v3.0.2 - quality assessment; Bowtie2 - read mapping; STAR v2.7 - RNASeq read mapping; Trinity v2.8.4 - transcript assembly; MAKER-P v2.31.10 - annotation; PASAv2.3.3 - annotation; RepeatModelerv1.0.11 - repeat annotation; OrthoFinder v2.2.7 - identify gene orthologues; CAFEv4.3.1 - gene family analyses; McScanX (version n/a)- synteny analyses; ClustalW v2.1 - sequence alignment; RAxMLv8.2.12 - phylogeny; RGAuguryv2017.10.21 - resistance gene annotation; BSMAPv2.9 - cytosine methylation; Nanopolish v0.10.1 - methylation analyses; EMBOSS v6.6.0.0 - CpG islands.

For manuscripts utilizing custom algorithms or software that are central to the research but not yet described in published literature, software must be made available to editors/reviewers. We strongly encourage code deposition in a community repository (e.g. GitHub). See the Nature Research guidelines for submitting code & software for further information.

Data

Policy information about availability of data

All manuscripts must include a data availability statement. This statement should provide the following information, where applicable:

- Accession codes, unique identifiers, or web links for publicly available datasets
- A list of figures that have associated raw data
- A description of any restrictions on data availability

Genome assembly and annotation, DNA-seq data, RNA-seq data and WGBS-seq data has been deposited to NCBI under BioProject ID PRJNA516907. A Jbrowse instance for each genome can be accessed at http://cruciferseq.ca. The YZ12151 B. nigra and B. rapa version 3 genome files were downloaded from http://brassicadb.org/brad/datasets/pub/Genomes. UniProt Release 2016_03 and Pfam32.0 were used in gene annotation and classification.

Field-specific reporting

Please select the one below that is the best fit for your research. If you are not sure, read the appropriate sections before making your selection.

☒ Life sciences ☐ Behavioural & social sciences ☐ Ecological, evolutionary & environmental sciences

For a reference copy of the document with all sections, see nature.com/documents/nr-reporting-summary-flat.pdf

Life sciences study design

All studies must disclose on these points even when the disclosure is negative.

Sample size	Sample size is not relevant, since reference genome sequences were being generated from representative samples of a specific species.
Data exclusions	No data was excluded from the analyses
Replication	Replication was limited but used where necessary to ensure the relevance of any analyses, for example bootstrapping in phylogenetic analyses, where at least 100 replications were used.
Randomization	Randomization was not relevant in the context of the types of statistical analyses performed, where predominantly genomic features or expression data was being analysed. There were no field or lab based experiment that might require randomization.
Blinding	There were no group comparisons performed, so blinding is not relevant.

Reporting for specific materials, systems and methods

We require information from authors about some types of materials, experimental systems and methods used in many studies. Here, indicate whether each material, system or method listed is relevant to your study. If you are not sure if a list item applies to your research, read the appropriate section before selecting a response.

Materials & experimental systems

n/a	Involved in the study	
☒	☐	Antibodies
☒	☐	Eukaryotic cell lines
☒	☐	Palaeontology
☒	☐	Animals and other organisms
☒	☐	Human research participants
☒	☐	Clinical data

Methods

n/a	Involved in the study	
☒	☐	ChIP-seq
☒	☐	Flow cytometry
☒	☐	MRI-based neuroimaging
